# Octameric dGTPase assemblies mediate broad anti-phage defense

**DOI:** 10.1038/s41467-026-75805-z

**Published:** 2026-07-25

**Authors:** Miao Shi, Peipei Li, Quanjin Li, Chao Ning, Sen Yin, Yina Gao, Yong Wang, Jingyu Li, Zhaolong Li, Songqing Liu, Dong Li, Changwei Wei, Ang Gao

**Affiliations:** 1 Medical Science and Technology Innovation Center, Shandong First Medical University & Shandong Academy of Medical Sciences, Jinan, China; 2 National Laboratory of Biomacromolecules, CAS Center for Excellence in Biomacromolecules, Institute of Biophysics, Chinese Academy of Sciences, Beijing, China; 3 University of Chinese Academy of Sciences, Beijing, China; 4 Key Laboratory of Molecular Medicine and Biotherapy, Aerospace Center Hospital, School of Life Science, Beijing Institute of Technology, Beijing, China; 5 State Key Laboratory of Membrane Biology, Tsinghua-Peking Center for Life Sciences, Beijing Frontier Research Center for Biological Structure, IDG/McGovern Institute for Brain Research, New Cornerstone Science Laboratory, School of Life Sciences, Tsinghua University, Beijing, China; 6 Anesthesia & Operation Center, Beijing Chao-Yang Hospital, Capital Medical University, Beijing, China; 7 Beijing Key Laboratory of Precision Translational Medicine in Anesthesiology and Pain, Beijing, China

**Keywords:** Cryoelectron microscopy, Bacterial structural biology, Proteins, Molecular biophysics

## Abstract

Deoxyguanosine triphosphatases (dGTPases) are nucleotide-depleting enzymes known to play a role in antiviral defense. While their enzymatic mechanism is established, the structural and functional diversity of dGTPases remains poorly understood. Here, we report a systematic analysis of dGTPase homologs across bacteria, archaea, and eukaryotes, revealing their widespread distribution and association with diverse immune-related domains. Through integrative bioinformatics and structural mining, we identify a class of bacterial dGTPases that assemble into stable octameric and higher-order oligomeric structures. Using cryo-electron microscopy, we resolve the octameric and 16-mer assemblies of a representative Vibrio dGTPase (Vdg) and further captured filamentous forms. Functional assays demonstrate that octamer formation is essential and sufficient for antiviral activity, while higher-order assemblies are dispensable. We also identify dAMP as an allosteric regulator, underscoring the functional versatility of dGTPases. Our findings provide insights into the modular architecture, oligomerization-driven activation, and immune function of bacterial dGTPases, and broaden our understanding of nucleotide depletion-based antiviral strategies.

## Abstract (web summary)

Deoxyguanosine triphosphatases (dGTPases) are nucleotide-depleting enzymes that contribute to antiviral defense. Here, the authors reveal widespread and structurally diverse dGTPases and characterize oligomeric dGTPases in distinct assembly states, providing insights into their architecture, activation, and regulation.

## Introduction

Bacteria deploy a remarkably diverse arsenal of defense systems to counter bacteriophage infection, many of which function by degrading essential components required for phage replication^1–3^. By interfering with key replication processes such as DNA synthesis, transcription, translation, and energy metabolism, these systems can effectively arrest the phage life cycle. Among them, classical systems such as restriction modification (RM) systems^4^, clustered regularly interspaced short palindromic repeats (CRISPR)-Cas systems^5,6^, achieve this by recognizing and cleaving foreign nucleic acids, while more recently identified systems such as Septu^7^ and Hachiman^8,9^, further expand this repertoire by targeting phage DNA to prevent the formation of viable progeny. Beyond genome targeting, certain systems inhibit viral protein synthesis. For instance, the PrrC protein^10,11^, and the PARIS defense system^12^ suppress translation through the cleavage of host tRNAs, effectively stalling phage replication. In parallel, several defense mechanisms act by perturbing host metabolic processes to induce abortive infection. The DSR2 systems, for instance, deplete intracellular NAD⁺, triggering an energetic collapse that arrests phage development progeny^13–15^. Given that phage replication is highly energy-intensive, some bacterial systems have evolved to directly target the energy pool. Detocs systems hydrolyze ATP by cleaving the N-glycosidic bond between the adenine base and ribose sugar, leading to rapid ATP depletion and the shutdown of viral replication^16^. Collectively, these component-degradation strategies underscore the evolutionary complexity of bacterial innate immunity and reveal how targeting the molecular infrastructure required for viral propagation forms a central axis of antiphage defense.

Deoxynucleoside triphosphates (dNTPs), which serve as the fundamental substrates for DNA synthesis and repair, play a pivotal role in preserving genome integrity and ensuring cell viability. The tight regulation of intracellular dNTP pools is crucial to maintain genomic stability and support normal cellular function^17^. Upon bacteriophage infection, host cells must preserve adequate dNTP levels to ensure the integrity of their own genomes and sustain cellular metabolism. Simultaneously, phage replication exerts significant pressure on these nucleotide pools, as viruses depend heavily on the host’s dNTP supply to drive viral genome replication and produce progeny. To restrict phage proliferation, bacteria have evolved a range of defense strategies that act by depleting intracellular dNTPs, thereby limiting the availability of substrates required for viral DNA replication. For instance, upon phage stimulation, the Cap17 protein cleaves deoxyadenosine triphosphate (dATP) into adenine and deoxyribose-5’-triphosphate, a reaction that effectively reduces the cellular pool of dATP necessary for phage genome synthesis^16^. Similarly, the AvcID system employs a deoxycytidine triphosphate (dCTP) deaminase that converts dCTP ultimately into deoxyuridine monophosphate (dUMP), limiting the availability of cytidine nucleotides^18^. In parallel, deoxyguanosine triphosphatases (dGTPases) hydrolyze deoxyguanosine triphosphate (dGTP) into deoxyguanosine (dG), further restricting cellular nucleotide pools^19–22^. Together, the strategy of depleting intracellular dNTP pools to inhibit viral replication illustrates the convergence of bacterial antiviral defense mechanisms.

An enzyme functionally analogous to bacterial dNTPases has also been identified in eukaryotic systems, most notably the antiviral restriction factor SAMHD1, which inhibits viral replication by hydrolyzing intracellular dNTPs, thereby depleting the nucleotide pools required for viral genome synthesis^23–25^. The presence of such dNTP-degrading enzymes across both prokaryotic and eukaryotic domains suggests that targeted nucleotide depletion represents a conserved strategy by which diverse organisms mount innate immune responses against viral pathogens. Although recent advances have expanded our understanding of bacterial dNTPase-mediated immunity, particularly through the identification of dGTPases with broad phylogenetic distribution among bacterial lineages^19^, important questions remain regarding the structural diversity, evolutionary origin, and precise mechanisms by which these enzymes contribute to antiphage defense. For example, while current structural studies have shown that bacterial dGTPases typically function as dimers that assemble into higher-order oligomers such as dimer^26^, tetramers^27,28^, or hexamers^20,21^, it is not yet clear whether additional modes of oligomerization exist, or whether such structural variation is mechanistically linked to phage resistance. These unresolved issues highlight the need for systematic, cross-kingdom investigation of dGTPase families, in order to define their molecular architectures, evolutionary conservation, and functional roles in nucleic acid-based immunity.

Here, using the Non-Redundant Protein Sequence Database, we systematically mined dGTPase homologs and discovered that dGTPase domains are broadly distributed across archaea, bacteria, and eukaryotes, suggesting that this enzyme family is both ancient and evolutionarily conserved. Notably, we observe that a subset of dGTPase proteins is fused with canonical immune-associated domains, such as caspase recruitment domains (CARD), death domains, and Toll/Interleukin-1 receptor (TIR) domains, suggesting that these enzymes may be functionally integrated into diverse immune signalling pathways. To explore the structural heterogeneity of these proteins, we employ a combined strategy involving Foldseek and AlphaFold-based modelling, through which we identified a previously uncharacterized oligomeric architecture of dGTPase that differs from all currently known configurations. This structural prediction was further supported by electron microscopy, which revealed the presence of a high-order octameric assembly. By combining biochemical and mutagenesis studies, we confirmed its anti-phage function. Together, our evolutionary, structural, and functional analyses define an octameric dGTPase and provide a repertoire in bacterial immunity.

## Results

### Diversity of dGTPase-containing proteins across archaea, bacteria and eukaryotes

dGTPases play a key component in antiviral immunity, frequently acting as effectors that deplete intracellular dGTP pools to inhibit phage replication. Initially identified in *Escherichia coli*, bacterial dGTPase contributes to abortive infection defense by hydrolyzing dGTP into deoxyguanosine, thereby limiting the substrates required for viral DNA synthesis^29–31^. A notable eukaryotic counterpart is human SAMHD1 enzyme, which contains both the dGTPase domain and a SAM domain and restricts viral replication through dNTP depletion^23–25^. Interestingly, while the SAM domain is dispensable for the catalytic activity of human SAMHD1, its conservation suggests potential regulatory or interaction roles. This raises the broader possibility that dGTPase domains may function in coordination with other protein modules, particularly within immune signaling pathways.

**Fig. 1:**
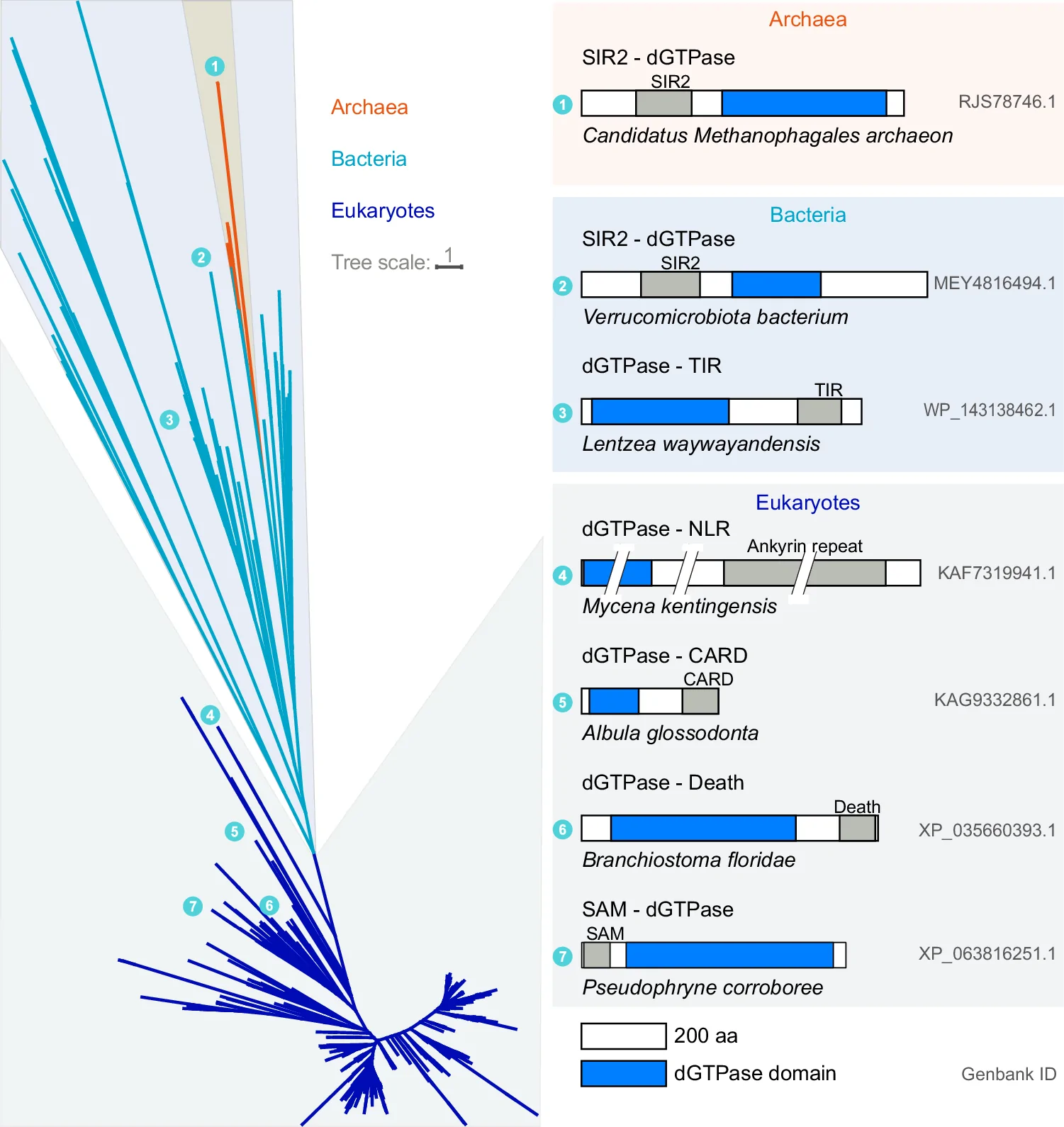
Diversity and domain architecture of immune proteins containing dGTPase domains. Left: Phylogenetic tree of dGTPase-containing proteins across archaea, bacteria, and eukaryotes, clustered based on sequence similarity of the core dGTPase domain. Right: Domain architecture of selected dGTPase-associated proteins, highlighting immune-related modules across different kingdoms. Abbreviations: SIR2 sirtuin, TIR toll/interleukin-1 receptor domain, CARD caspase recruitment domain, NLR nucleotide-binding leucine-rich repeat domain, SAM Sterile alpha motif domain.

To explore this, we systematically mined the Non-Redundant Protein Sequence Database (NR) database for proteins harboring dGTPase domains. We identified 950 non-redundant proteins containing dGTPase as a core domain, spanning all three domains of life: archaea, bacteria, and eukaryotes (Fig. 1). Notably, eukaryotes exhibit a disproportionately high abundance of dGTPase-containing proteins (92%) relative to bacteria (7.47%) and archaea (0.53%) (Supplementary Fig. 1a), indicating broad evolutionary conservation and potential functional diversification.

Domain architecture analysis reveals that dGTPase domains are frequently fused to other modules implicated in immunity and inflammation, including SAM domains (as in SAMHD1), nucleotide-binding leucine-rich repeat receptors (NLRs), Toll/Interleukin-1 receptor (TIR) domains, as well as three additional well-characterized immune-related domains (Fig. 1). In NLR-like architectures, the dGTPase domain is positioned at the N-terminus, a region typically associated with effector function in innate immunity. Given that the C-terminus of NLRs often serves as a pathogen-sensing module, the domain arrangement suggests that dGTPase may act as a functional effector that executes the immune response following pathogen recognition. Similarly, we identified fusions of dGTPase domains with the TIR domain, canonical signaling modules that initiate immune responses in both eukaryotes and prokaryotes^32–35^. In such architectures, the TIR domain likely functions as a pathogen sensor or signal transducer, while the dGTPase domain may serve as the downstream effector that executes cellular responses, such as abortive infection via nucleotide depletion.

We also identified dGTPase domains fused to other immune-associated modules in diverse species. For instance, in the metazoan *Branchiostoma floridae*, dGTPase is fused with a death domain, which is typically involved in inflammasome assembly and apoptotic signaling^36,37^. In *Candidatus Methanophagales* archaeon and *Verrucomicrobiota* bacteria, dGTPase is fused to a SIR2-like domain, potentially replacing the SLOG-like domain of ThsA proteins in NAD-dependent antiphage systems^38^. This substitution may facilitate oligomeric rearrangements or enzymatic activation. Collectively, these findings suggest that dGTPases function as versatile immune effectors, frequently co-opted into diverse host defense architectures to mediate antiviral activity through targeted nucleotide depletion.

### Effector dGTPase associated with higher-order oligomeric assemblies

In our analysis of dGTPase-related defense systems, we observed that dGTPase can sometimes occupy positions analogous to domains known for their aggregation potential. For example, dGTPase is found coupled with the TIR domain, whereas STING domain- and SAVED domain-containing proteins are also commonly associated with TIR domains. These STING /SAVED-TIR assemblies have been shown to mediate filament formation and possess antiviral activity^39,40^. Based on these observations, we propose that dGTPase may serve as an analogous module to these aggregation-prone domains in certain immune contexts. Moreover, in many of the identified domain architectures, dGTPase appears to serve primarily as an effector module acting downstream of sensor or signaling components such as TIR^40^ or NLR-like domains^41^. Notably, a variety of immune effectors are known to function through oligomerization or polymerization, forming higher-order assemblies to amplify or execute their activity^42–44^. Based on these observations, we hypothesized that dGTPase might likewise leverage polymerization as a mechanism to exert its effector function.

To assess this possibility, we first reviewed available structural data and noted that previously reported dGTPase structures predominantly adopt dimer^26^, tetrameric^27^, or hexameric^20^ configurations. While these forms suggest a degree of oligomerization, they do not fully account for the effector-like properties typically associated with large polymeric assemblies. We therefore posited that alternative, higher-order oligomeric states of dGTPase may exist and contribute to its immune function. Notably, our bioinformatic analysis revealed that proteins containing only the dGTPase domain are approximately six times more abundant than those fused to other immune-related domains (Supplementary Fig. 1b). Given their prevalence, we therefore focus this study on these standalone dGTPase proteins.

To explore this, we conducted structural mining within the AlphaFold Database (AFDB), using the structural features of the core dGTPase domain of human SAMHD1 as a reference model. A monomeric dGTPase structure was first constructed and used to query potential homologs across diverse species. From this, we identified 43 candidate proteins with dGTPase domains. Remarkably, structural predictions revealed that several of these candidates, most notably from *Pseudomonas aeruginosa* PAO1, exhibited a distinctive square-shaped octameric conformation, despite moderate predicted confidence scores (e.g., iPTM score of 0.59).

**Fig. 2:**
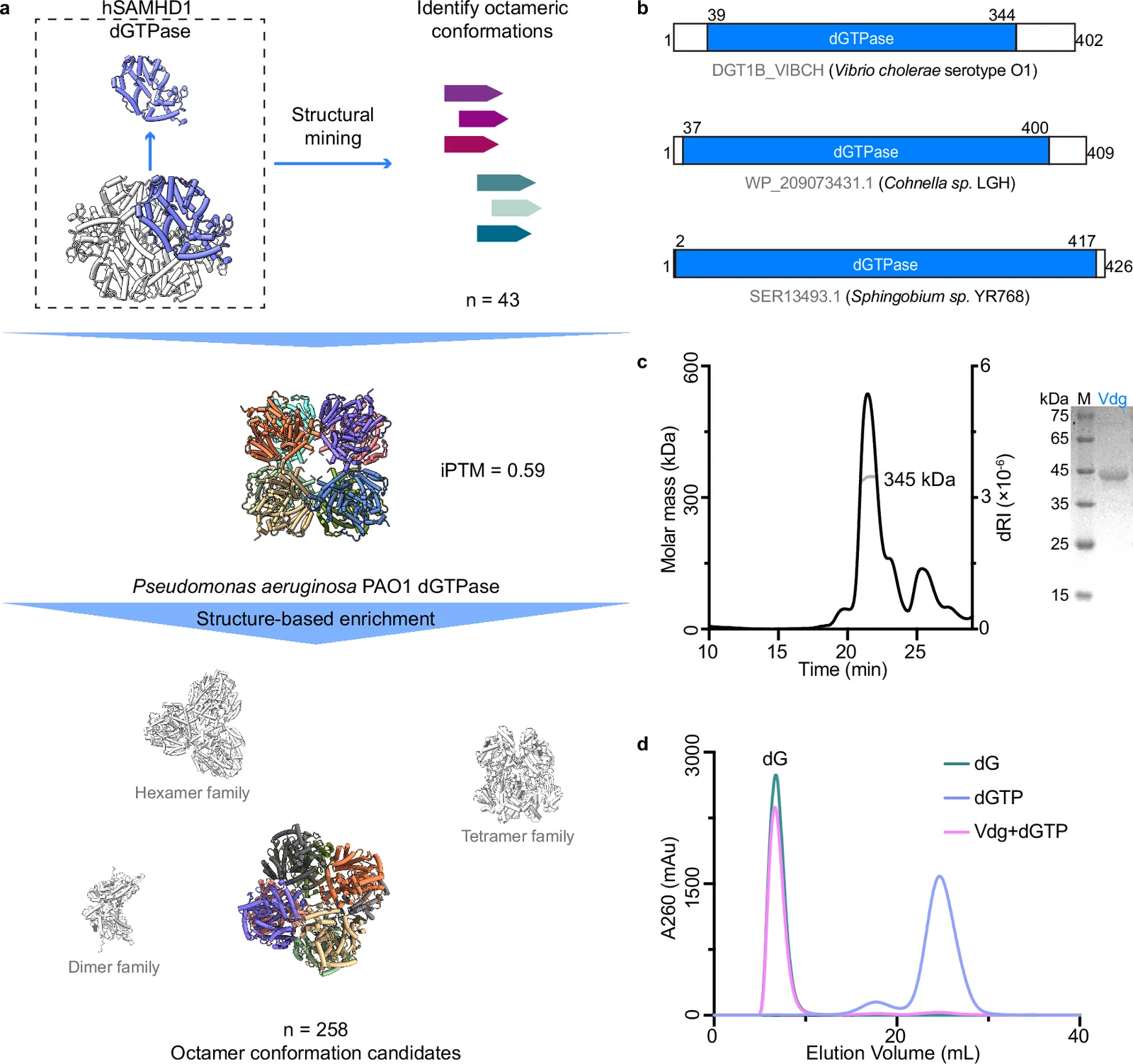
Discovery and structural prediction of oligomeric dGTPase systems. **a** Structural mining and identification of oligomeric dGTPase systems. The dGTPase domain of human SAMHD1 (PDB: 4bzb, residues 129–439) was used as a seed to identify structural homologs of dGTPase proteins. Iterative structure-based mining identified candidate proteins with predicted octameric architectures, including a representative from *Pseudomonas aeruginosa* PAO1 (iPTM = 0.59). High-confidence candidates were further filtered using the AlphaFold Database (AFDB), leading to the identification of putative octameric assemblies. **b** Schematic representation of selected dGTPase systems from representative species, with domain architectures annotated and residue numbers indicated. Genbank accession numbers are shown in grey. Species included *Vibrio cholerae* serotype O1 (Genbank ID DGT1B_VIBCH); *Cohnella sp*. LGH (Genbank ID WP_209073431.1); *Sphingobium sp*. YR768 (Genbank ID SER13493.1). **c** Left: Schematic representations illustrating the expression strategies and size-exclusion chromatography coupled with multi-angle light scattering (SEC-MALS) analysis of Vdg. The gray line indicates the measured molecular mass (kDa), while the black line represents the protein concentration (dRI; change in refractive index from baseline). The measured molecular mass of the Vdg oligomer is approximately 345 kDa. Right: SDS-PAGE analysis confirming the purity and integrity of the Vdg protein. Data are representative of at least three experiments. **d** In vitro dGTP hydrolysis assay demonstrates that Vdg possesses dGTPase activity, as evidenced by substrate depletion upon incubation with purified protein. The dG standard is colored green, while the dGTP standard is colored purple.

Using this unique 3D structural feature as a template and the Foldseek server as a rapid structural search tool following AlphaFold 3 model generation, bioinformatic screening identified 258 of 792 dGTPase family members (iPTM > 0.6) that are predicted to adopt this previously uncharacterized octameric assembly (Fig. 2a and Supplementary Fig. 1c). This finding suggests that dGTPases possess a greater degree of structural plasticity than previously appreciated and that such alternative oligomeric forms may play functional roles in immune effector activity in previously unexplored ways, particularly in the context of bacterial antiphage defense.

### dGTPase forms an oligomeric octamer and filament-like assemblies

To investigate the oligomeric organization of dGTPase, we selected three representative species, *Vibrio cholerae* serotype O1, *Cohnella sp*. LGH, and *Sphingobium sp*. YR768, based on their high-confidence AlphaFold structural predictions (iPTM scores up to 0.88) (Fig. 2b). Despite considerable sequence diversities, all three proteins share a conserved dGTP triphosphate hydrolase domain, suggesting functional conservation across species.

To biochemically characterize the oligomeric state and catalytic activity, we focused on the dGTPase from *Vibrio cholerae* serotype O1, which we renamed Vdg. Vdg was recombinantly expressed in *E. coli* BL21(DE3) and successfully purified to homogeneity and appears to assemble into an octameric complex (Fig. 2c), enabling downstream structural and enzymatic analyses.

We first examined the in vitro enzymatic activity of Vdg. Purified protein was incubated with dGTP, and the product formation was analyzed using Resource Q chromatography. The reaction showed substantial depletion of dGTP compared to control samples lacking Vdg, confirming that Vdg possesses dGTP triphosphatase activity in vitro (Fig. 2d).

**Fig. 3:**
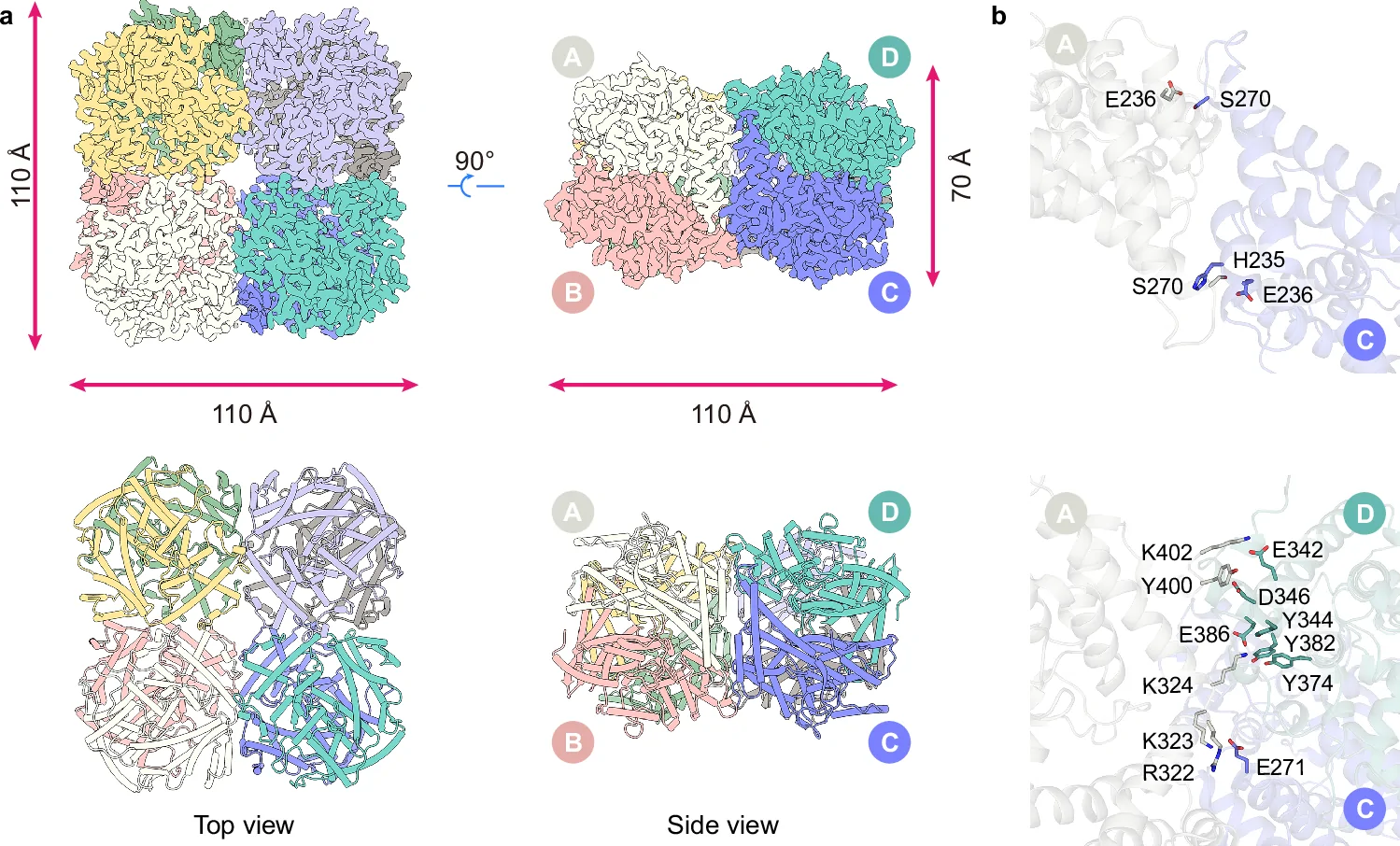
The overall structure of Vdg octamer. **a** Top: Cryo-EM density map of Vdg octamer, with each subunit shown in a distinct color. The complex adopts a square-shaped architecture with approximate dimensions of 110 Å by 110 Å by 70 Å. Bottom: Cylinder representation of the Vdg octamer, with subunits colored to correspond to the cryo-EM density map. **b** Inter-subunit interactions mediating assembly of the Vdg octamer. Key residues located at the dimer-dimer interface are shown in stick representation, illustrating the contacts that stabilize higher-order oligomerization.

To determine the oligomeric architecture of Vdg, we determined its structure using cryo-EM at a resolution of 3.16 Å (Fig. 3a, Supplementary Fig. 2a–d and Supplementary Table 1), allowing de novo atomic model building. The assembled complex exhibits overall dimensions of 110 × 110 × 70 Å (Fig. 3a). Unlike previously reported bacterial dGTPases, which primarily form tetramers or hexamers^20,27^, Vdg assembles into a strikingly symmetric square-shaped octamer, composed of four dimers arranged in a quadrangular configuration. Interactions between four dimers stabilize the octameric assembly. Specifically, the side chain of E236, along with the backbone atoms of A264 and S270 from chain A, interacts with S270, H235, and E236 of chain C, respectively. Additionally, R322 and K323 from chain A form hydrogen bonds with E271 of chain C. K324 further establishes hydrogen-bonding interactions with Y344, Y374, Y382, and E386 of chain D. At the C-terminus, residues Y400 and K402 of chain A engage with D346 and E342 interactions (Fig. 3b and Supplementary Fig. 2e). These extensive inter-chain interactions, which connect the four dimers within the octamer, are important for enzymatic activity (Supplementary Fig. 2f).

### Structural basis for substrate recognition and allosteric activation of Vdg

Having established that Vdg assembles into an octameric structure with dGTPase activity, we next sought to dissect the molecular mechanisms underlying substrate recognition and allosteric regulation. Previous studies have demonstrated that dGTPase activity can be modulated by allosteric ligands such as dATP^21^, prompting us to investigate the structural basis underlying its enzymatic activity and allosteric regulation^21^.

To stabilize the precatalytic state and capture substrate-bound structures, we introduced a point mutation at the putative active site. Sequence alignment identified His114, part of a proposed catalytic dyad (His114/Glu117)^22,45^, as a universally conserved residue in the dGTPase family (Supplementary Fig. 3a). Analogous mutations at the corresponding histidine residues in *E. coli*, *L. blandensis*, and human SAMHD1 have been shown to significantly reduce enzymatic activity^21,22,45^. Consistently, the H114A mutant of Vdg exhibited a strong reduction in dGTPase activity (Supplementary Fig. 3b, c), enabling us to capture substrate-bound conformations for structural analysis.

**Fig. 4:**
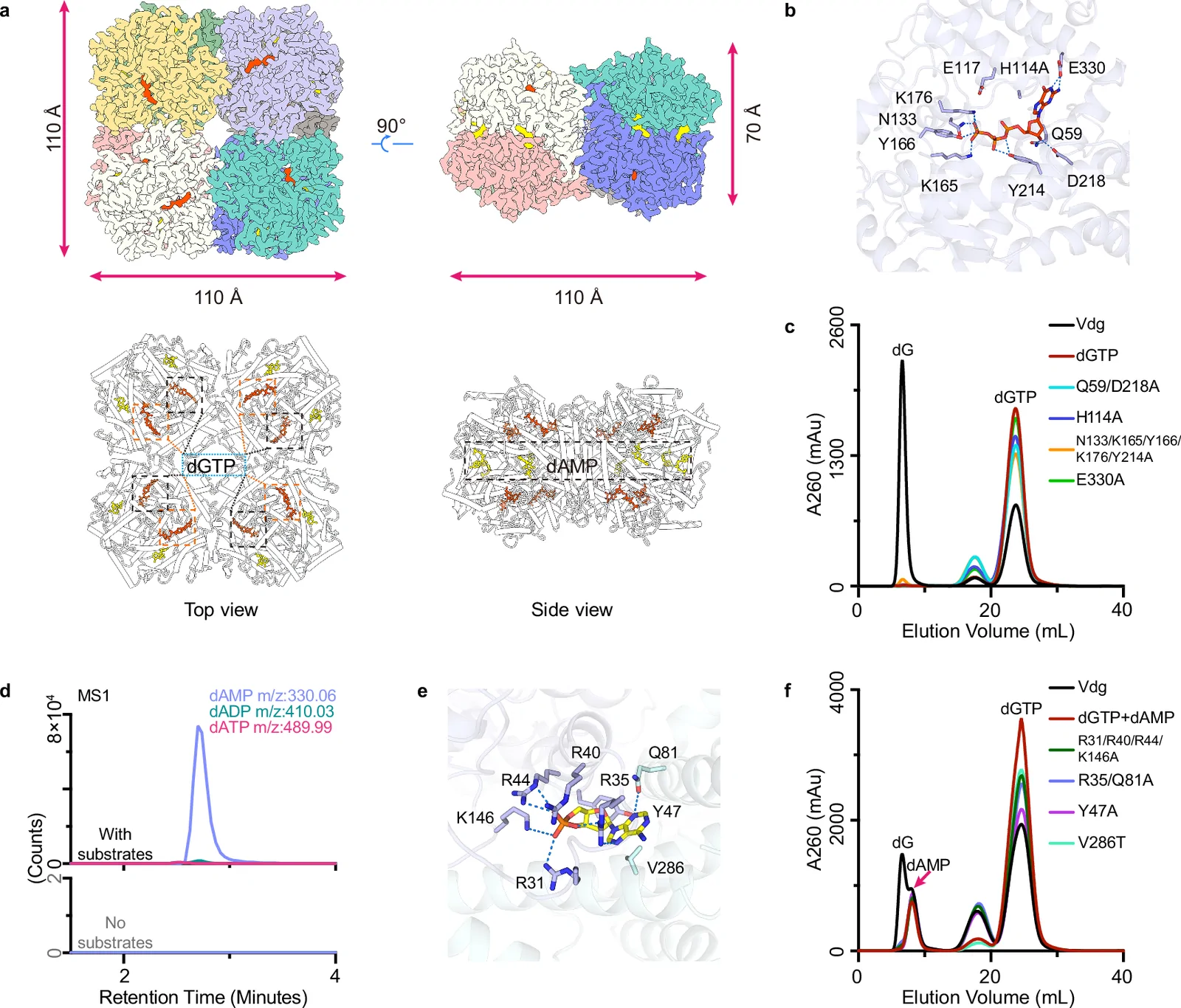
Cryo-EM structure of Vdg H114A bound to dGTP and dAMP. **a** Top: Cryo-EM density map of the catalytically impaired Vdg H114A octamer in complex with dGTP and dAMP. Bottom: Cylinder representation of the Vdg H114A octamer, colored by subunit. Eight dGTP and eight dAMP molecules (ball-and-stick) are positioned at the active and allosteric sites, respectively. **b** Key residues coordinating dGTP within the active site. Hydrogen bonds are shown as blue dashed lines. **c** In vitro chromatograph assays of wild-type Vdg and mutations affecting the dGTP hydrolysis. Data are representative of at least three experiments. **d** Mass spectrometry analysis confirms dAMP as the primary ligand bound to Vdg H114A, rather than dATP or dADP. Top: Following incubation of Vdg H114A with nucleotide substrates, unbound ligands were removed by size-exclusion chromatography. Mass spectrometry revealed that dAMP was the predominant bound ligand (7.5 × 10⁵), compared to much lower levels of dADP (2.1 × 10⁴) and dATP (7.1 × 10³). Bottom: No ligands were detected in control samples in the absence of added nucleotides. **e** Stick representation of residues coordinating dAMP at the allosteric site. The two Vdg subunits are colored purple and cyan, respectively. Hydrogen bonds are shown as blue dashed lines. **f** In vitro chromatography assays showing the impact of allosteric-site mutations on dGTP hydrolysis. Data are representative of at least three experiments.

We solved the high-resolution cryo-EM structure of to the catalytically impaired Vdg H114A mutant at resolution of 2.96 Å, revealing dGTP molecules bound at active sites and dAMP ligands occupying the allosteric activation sites (Fig. 4a, Supplementary Fig. 4a–c and Supplementary Table 1). The structure reveals clear density for all eight dGTP molecules, with each monomer in the octamer binding one substrate molecule (Fig. 4a and Supplementary Fig. 5a, b). Electrostatic surface analysis shows that dGTP is nestled within a deep binding pocket (Supplementary Fig. 5c), with its deoxyguanosine moiety stabilized by Q59, D218, E330, and the backbone carbonyl of L60, while the triphosphate tail is coordinated by positively charged residues including N133, K165, Y166, and K176 (Fig. 4b and Supplementary Fig. 5d). Y214, a universally conserved residue, contributes van der Waals contacts and interacts with the sugar and β-phosphate of the dGTP (Fig. 4b and Supplementary Fig. 5d). To validate the functional importance of these sites in dGTP hydrolysis, we generated mutations in key residues involved in dGTP coordination. Our results indicate that these dGTP-binding sites contribute to the enzymatic inactivity of Vdg (Fig. 4c).

Interestingly, although dATP is not a substrate for the Vdg (Supplementary Fig. 5e), chromatography and mass spectrometry reveal the presence of dATP hydrolysis products, notably dADP and dAMP. Cryo-EM structure of the Vdg-ligand complex showed that the allosteric site specifically accommodates dAMP, not dATP or dADP (Supplementary Fig. 5f, g). Structurally, this pocket is compatible with dAMP, whereas dADP or dATP would introduce additional phosphate groups that are incompatible with the observed pocket geometry and would cause steric clashes with surrounding residues, particularly R31 (Supplementary Fig. 5f, g). Accordingly, non-hydrolyzable dATP analogs, which retain these additional phosphate groups and cannot be converted into dAMP, would not be expected to induce the conformational changes associated with dAMP binding. To validate this observation, we purified Vdg and its ligands complex, followed by mass spectrometry analysis, which confirmed the binding of dAMP (Fig. 4d and Supplementary Fig. 5h). Notably, Vdg binds dAMP with levels 35-fold higher than dADP and undetectable in the absence of substrate (Fig. 4d). This indicates that dAMP, rather than dATP, acts as a physiological allosteric regulator of Vdg.

The dAMP-binding site is located at the monomer-monomer interface of each Vdg dimer, with two dAMP molecules bound symmetrically per dimer (Supplementary Fig. 5i, j). This position overlaps with known effector-binding sites in other dGTPases, including the ssDNA site in *E. coli* dGTPase and the dATP site in *L. blandensis*. dAMP is deeply buried in a positively charged pocket and stabilized by stacking interactions with R35 and Y47, as well as hydrogen bonds involving Q81 (via the Watson-Crick edge of the adenine base) and R31, R35, R40, R44 and K146 with the phosphate group (Fig. 4e, f and Supplementary Fig. 5k, l). The essential role of the residue V286 in dAMP recognition is further demonstrated by the V286T mutation, which compromises dGTPase activity (Fig. 4e, f and Supplementary Fig. 5k, l). The identified allosteric binding site for dAMP highlights the multifunctionality and evolutionary adaptability of the dGTPase domain.

**Fig. 5:**
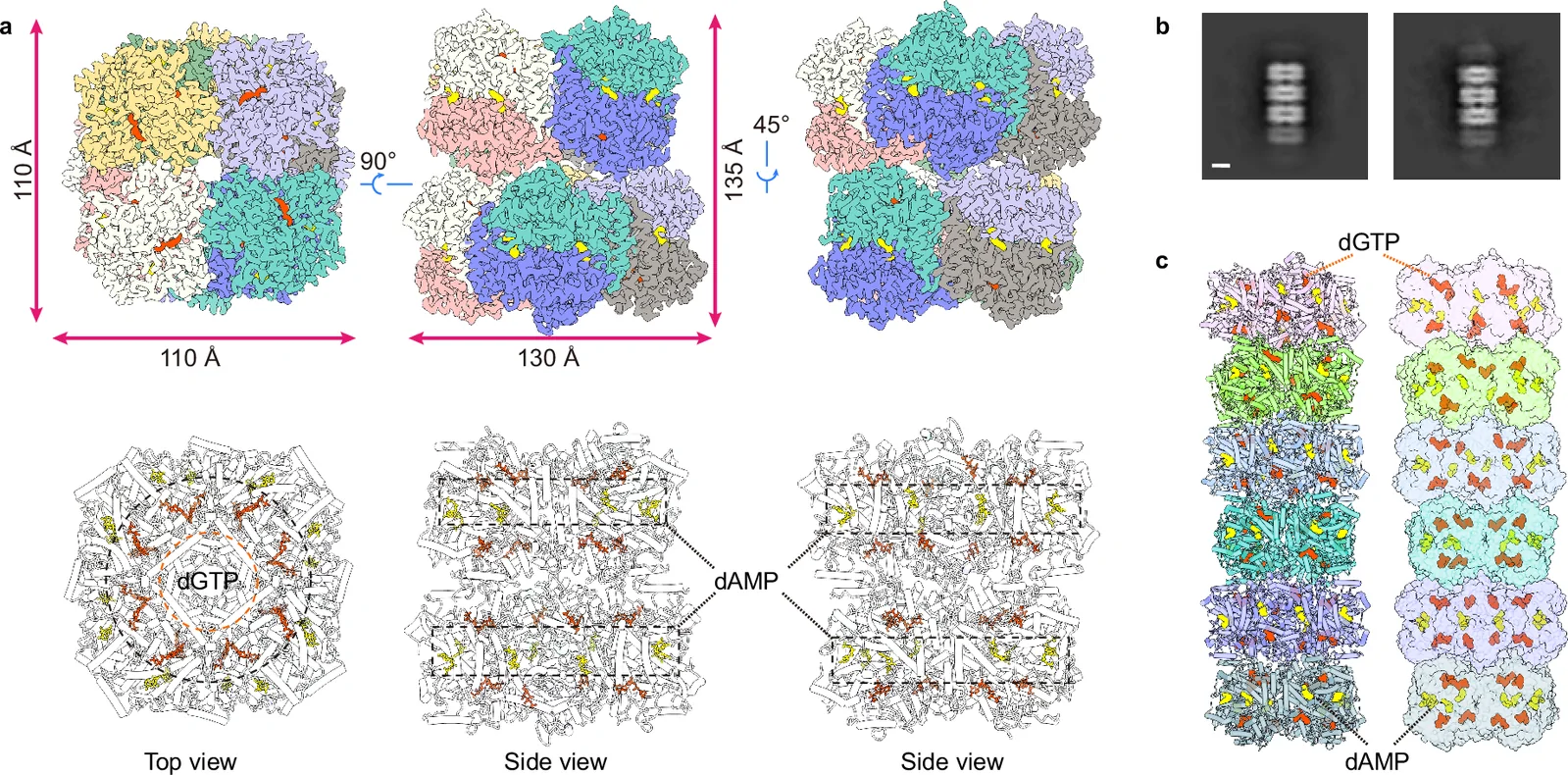
Cryo-EM structure of Vdg H114A 16-mer bound to dGTP and dAMP. **a** Top: Cryo-EM density map of 16-mer complex formed by two stacked octamers of Vdg H114A, each bound to dGTP and dAMP. Bottom: Cylinder representation of the 16-mer, with subunits colored to match the cryo-EM density map. Sixteen dGTP and sixteen dAMP molecules (shown as ball-stick models) are positioned at the active or allosteric sites. The two octamers are stacked with an ~45° rotation. **b** Representative 2D class averages from reference-free alignment, revealing filament-like assemblies formed by Vdg. Scale bar, 5 nm. **c** Ribbon (left) and surface (right) representations of a Vdg filament-like model bound to dGTP and dAMP.

Beyond the octameric structure, we further discovered a 16-mer assembly composed of two stacked octamers by cryo-EM at 3.29 Å resolution, highlighting the structural versatility of Vdg. In this higher-order assembly, the two stacked octamers are rotated approximately 45° relative to one another, forming a modular 16-subunit architecture (Fig. 5a and Supplementary Fig. 4a, d, e). The structure of the 16-mer is best described as a pair of parallel dimers with distinct angles of alignment.

In addition to the 16-mer, 2D class averaging revealed that Vdg can further organize into filament-like assemblies composed of stacked octameric units (Fig. 5b). These filaments were relatively sparse and exhibited considerable flexibility, which precluded atomic-resolution reconstruction. Nonetheless, their existence suggests that Vdg possesses an intrinsic capacity for higher-order polymerization (Fig. 5c), potentially enabling adaptor-like functions in signaling contexts. Compared to canonical immune filaments, the Vdg filaments appeared less stable or elongated, raising the possibility that Vdg represents either evolutionary intermediates bridging small oligomeric enzymes and large polymeric immune complexes, or transient/ regulatory assemblies formed under specific cellular conditions.

Structural inspection of both the 16-mer assemblies reveals hydrogen bond networks involving residues C127, C366, D180, and N364 located on the surface of different subunits from adjacent octamers. These interactions mediate both octamer-octamer contacts and support the formation of filamentous architectures (Supplementary Fig. 6a, b).

To evaluate the functional importance of residues involved in octameric and filamentous assembly, we generated the C127A/C366A and D180A/N364A mutants of Vdg. Chromatography analysis revealed that neither mutant significantly impaired Vdg dGTPase activity in vitro (Supplementary Fig. 6c). These findings suggest that while Vdg is capable of forming higher-order filamentous assemblies, such structures may not be required for its catalytic activity. Instead, the octameric conformation likely represents the core functional unit, with filament formation potentially representing a vestigial or regulatory feature in Vdg’s evolutionary trajectory.

### The anti-phage activity of Vdg

**Fig. 6:**
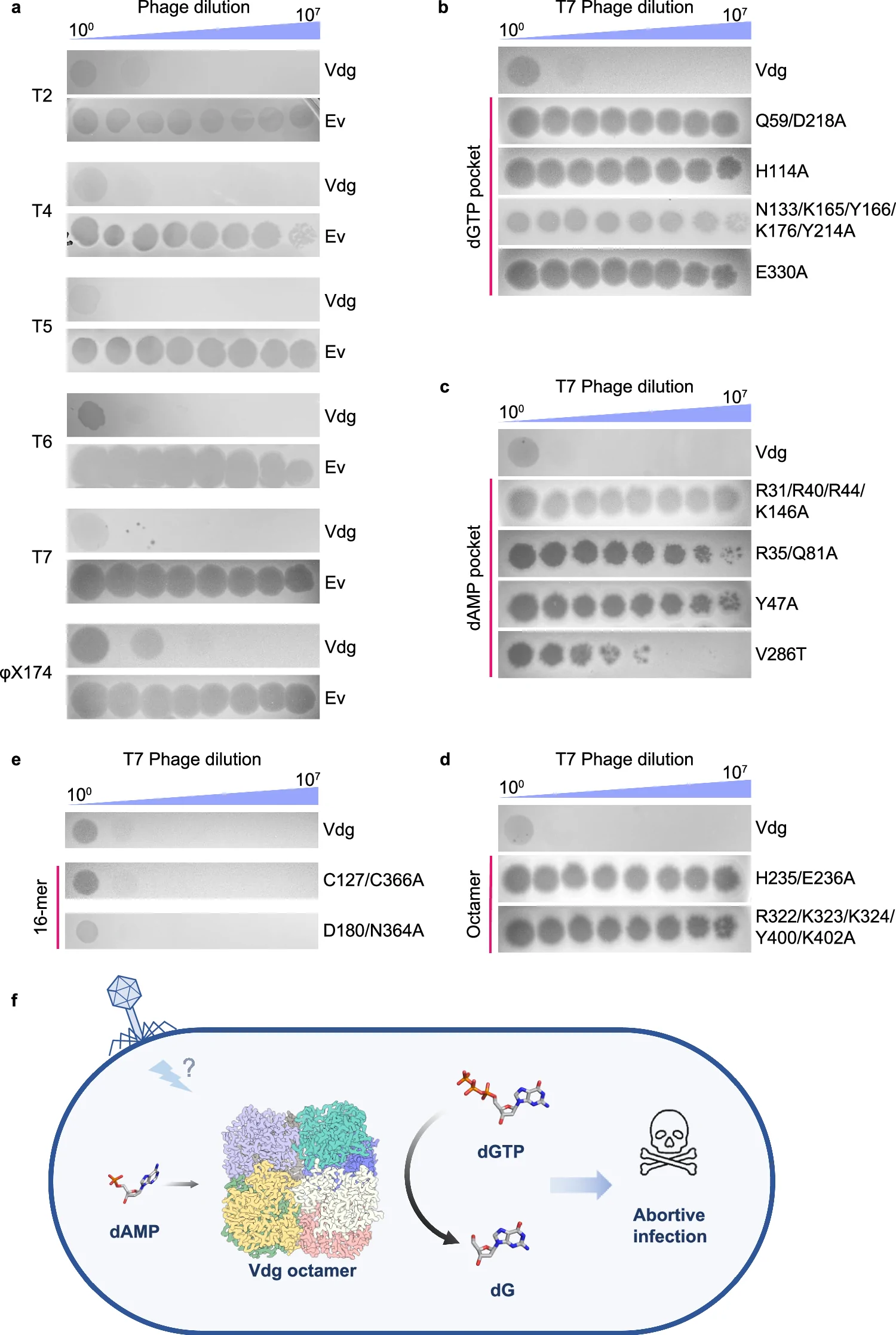
Anti-phage activity of the Vdg system. **a** Bacterial growth assays showing the protective effect of Vdg expression against infection by a panel of bacteriophages, including both ssDNA (ϕX174) and dsDNA (T2, T4, T5, T6, T7) phages. “Ev” indicates the empty vector control. **b**–**e** Bacterial growth assays assessing the antiphage activity of wild-type (WT) Vdg and various mutants upon T7 infection. **b** WT and mutants targeting the dGTP catalytic pocket. **c** WT and mutants targeting the allosteric binding site. **d** WT and mutants affecting the dimer to octamer interface. **e** WT and mutants disrupting the octamer-octamer interface. All data are representative of at least three independent experiments. **f** Working model of the Vdg anti-phage defense system.

To evaluate the anti-phage activity of Vdg, we tested its activity against a panel of bacteriophages with either single-stranded DNA (ssDNA; φ174) or double-stranded DNA (dsDNA; T2, T4, T5, T6, and T7) genomes. Compared to *E. coli* carrying an empty vector, *E. coli* expressing Vdg exhibits robust resistance to all tested phages, indicating broad-spectrum anti-phage activity (Fig. 6a). This inhibitory effect is likely attributable to enzymatic degradation of dGTP, thereby limiting the nucleotide pool required for phage genome replication. To confirm that this activity is dependent on the catalytic function of Vdg, we expressed the H114A mutant, previously shown to lack dGTPase activity, in *E. coli*. Cells expressing this mutant fail to suppress phage replication, underscoring the essential role of dGTPase activity in anti-phage defense (Fig. 6b). In addition, other mutants in dGTP or dAMP pockets, which have been validated to impair dGTP degradation activity, were also found to reduce anti-phage defense (Fig. 6b, c).

To examine the importance of octamer formation for the anti-phage function of Vdg, we introduced interface-disrupting mutations, H235/E236A and R322/K323/K324/Y400/K402A, at the dimer-to-octamer interface. These mutations severely impaired the antiviral activity of Vdg, highlighting the essential role of octamerization in its biological function (Fig. 6d). Next, we tested whether higher-order oligomeric forms, such as the 16-mer or filamentous assemblies, are required for anti-phage function. To this end, we introduced the C127/C366A and D180/N364A mutations (Supplementary Fig. 6a, b), which disrupt octamer-octamer interactions while preserving the core octamer structure. Both mutants retain anti-phage activity comparable to that of wild-type Vdg, consistent with in vitro data showing intact enzymatic function (Fig. 6e and Supplementary Fig. 6c). These results further support the notion that the octameric conformation is the minimal and sufficient functional unit of Vdg, while higher-order assemblies may serve regulatory or evolutionary roles.

Together with our bioinformatic, structural, and functional analyses, these findings support a model in which Vdg functions as an adaptor-like dGTPase effector that is capable of assembling into oligomeric complexes. In particular, we identified previously uncharacterized higher-order assemblies, including a 16-mer architecture and filament-like structures, which are structurally built upon the octameric scaffold of Vdg. Despite this structural versatility, the octameric conformation alone is sufficient to carry out the biological function of Vdg in dGTP degradation and antiphage defense. Collectively, our data suggest that Vdg plays a key role in bacterial immune defense against phage infection by depleting intracellular dGTP, thus triggering abortive infections.

## Discussion

Phage infections exert strong evolutionary pressure on host cells, leading to the development of a wide variety of anti-phage defense mechanisms. One such strategy involves the depletion of intracellular nucleotide pools to disrupt phage replication. dGTPases, which hydrolyze dGTP into deoxyguanosine and triphosphate, play a critical role in this process. Here, we reveal that dGTPase is a widespread and evolutionarily adaptable anti-phage effector, showing remarkable diversity in species distribution, domain architecture, and oligomeric organization.

Through comprehensive bioinformatic analyses, we identified nearly one thousand dGTPase-containing proteins spanning archaea, bacteria, and eukaryotes. Our bioinformatic survey showed that dGTPase domains are frequently coupled with immune-related domains such as TIR, NLR, CARD, and SIR2. Such domain architectures suggest that dGTPase acts as an enzymatic effector coupled to pathogen recognition modules, operating within an ancient immune framework. We further identified a class of dGTPases predicted to adopt an octameric conformation (iPTM > 0.6) through structure-based mining and AlphaFold 3 predictions. Structural experiments provide evidence for the existence of octameric dGTPases. However, not all high-confidence predictions necessarily correspond to stable octameric assemblies, and experimental validation will be required to confirm their oligomeric states.

Structurally, we uncovered a previously uncharacterized oligomeric form of dGTPase. Although earlier work described dimeric, tetrameric, and hexameric dGTPase conformations, we identified an octameric assembly that is functionally sufficient for dGTP hydrolysis and antiphage defense. To place this architecture in context, we compared Vdg with representative dGTPases of different oligomeric states. The Vdg structure contains eight subunits organized as four dimers, with each monomer harboring one catalytic pocket. By comparison, the hexameric dGTPase from *Leeuwenhoekiella blandensis* (PDB: 7TU5 and 7TU6) contains six subunits organized as three dimers, whereas the dNTPase from *Enterococcus faecalis* has been observed in both dimeric and tetrameric forms (Supplementary Fig. 6d). At the level of the dimeric building block, Vdg is most similar to the hexameric dGTPase: the dimers can be well superimposed (RMSD = 2.478 Å) and share highly similar monomer-monomer interfaces, allosteric sites, and catalytic pockets (Supplementary Fig. 6e). However, the higher-order assemblies differ in how these dimers pack against one another. In Vdg, two helices at the inter-dimer interface adopt markedly different orientations, with angular displacements of 72.4° and 60.11°, respectively, thereby generating additional dimer-dimer contacts that support the octameric assembly (Supplementary Fig. 6f). Together, these observations highlight both the conserved dimeric core and the diversified higher-order architectures of antiviral dGTPases.

Beyond the core octameric form, we also observed higher-order assemblies, including a 16-mer composed of two stacked octamers and filament-like structures formed by linear octamer stacking. However, mutational analysis showed that disrupting the interfaces involved in higher-order assembly did not impair dGTPase activity or phage resistance, indicating that the octamer alone is the key functional unit. These additional assemblies may represent evolutionary intermediates or transient states with potential regulatory roles.

We also discovered that dAMP serves as an allosteric ligand for Vdg at a conserved site between dimers. Since dAMP binds at the monomer-monomer interface, mutations at the dimer-dimer interface do not affect dAMP binding. The binding of dAMP can be detected by mass spectrometry analysis. (Supplementary Fig. 6g)^21, 26^. Given the structural similarity to known allosteric sites in related systems (e.g., SAMHD1, Ec-dGTPase), it is likely that small nucleotide-like ligands may serve as regulatory signals for dGTPase activation in response to infection. Although dAMP acts as an allosteric activator of the dGTPase, there is currently no direct evidence regarding the temporal order of dAMP and dGTP binding or release. Importantly, the enzyme retains catalytic activity in the absence of dAMP, indicating that dAMP is not absolutely required for catalysis but instead enhances dGTPase activity when present. Therefore, while a downstream relationship between dGTP binding/hydrolysis and dAMP binding remains possible, the current data support a model in which dAMP functions as an allosteric enhancer rather than an essential prerequisite for enzymatic activity. Taken together, our findings support a model in which Vdg functions as an oligomeric dGTPase effector that can assemble into higher-order structures but relies primarily on its octameric form for enzymatic and antiviral activity (Figs. 5c and 6f). In detail, the current structural and biochemical data allow us to propose a mechanistic model for allosteric activation of Vdg by dAMP (Supplementary Fig. 6h). In this model, dAMP binding induces a rotation of chain D by 4.57° relative to chain A, while chain C undergoes a larger rotation of 5.84° relative to chain A, resulting in an overall shift at the dimer-dimer interface (Supplementary Fig. 6h). This allosteric effect of dAMP binding is further transmitted to a critical substrate-binding loop (Supplementary Fig. 6h), supporting structural crosstalk between the allosteric and catalytic sites. At each monomer-monomer (intradimer) interface, there are two dAMP-binding sites (denoted dAMP1 and dAMP2). The conformational changes observed in the dAMP-bound structures suggest distinct roles for each dAMP molecule. Specifically, dAMP1 appears to influence the active site of the same monomer, whereas dAMP2 primarily affects the active site of the adjacent monomer within the dimer. Upon dAMP binding, the loops containing R35 push against the helix connected to the substrate-binding loop, thereby promoting closure of the dGTP-binding pocket. A complementary effect is exerted by dAMP2 on the adjacent monomer within the dimer. These results expand our understanding of how dGTPase-mediated nucleotide depletion contributes to antiphage immunity and highlight the structural plasticity of this enzyme family.

In conclusion, our study uncovers an octamer-based mechanism for dGTP depletion in antiviral defense, expands the known structural repertoire of the dGTPase family, and highlights the evolutionary plasticity as immune effectors. These findings provide a framework for future exploration of nucleotide-depletion strategies in both prokaryotic and eukaryotic systems, and open avenues for discovering defense mechanisms built on similar enzymatic platforms.

## Methods

### Identification of dGTPase-associated protein sequences from the NR database

To comprehensively identify protein sequences related to dGTPase, we performed a reverse BLAST search using the Non-Redundant Protein Sequence Database (NR) database as the query and the dGTPase sequence as the subject database. This approach was selected to minimize the potential bias introduced by the random order of conventional BLAST searches, and to ensure the retrieval of sequences with significant homology to dGTPase. The search was carried out using BLAST+ (version 2.12.0+) with the blastp algorithm under the following parameters:

blastp -query NR -db dGTPase -out NR2dGTPase -num_threads 10 -outfmt ‘6 qlen slen qseqid sseqid pident length mismatch gapopen qstart qend sstart send evalue bitscore’ -max_target_seqs 5 -evalue 1e-2. To remove abnormally long or likely artifactual sequences, we filtered out hits with protein lengths exceeding 20,000 amino acids.

### Domain annotation of candidate sequences

All retained sequences were subjected to large-scale protein domain annotation using InterProScan (version 5.69-101.0). To maximize annotation coverage and functional information, we enabled multiple domain databases, including Pfam, PRINTS, PANTHER, ProSiteProfiles, and SMART. InterProScan was run with Gene Ontology and InterPro annotation options enabled, using the following command: interproscan.sh -iprlookup -goterms -appl Pfam -appl PRINTS -appl PANTHER -appl ProSiteProfiles -appl SMART -f TSV -i input.fasta -o output.ipscan. Based on the annotated results, proteins were retained for further analysis only if they either (i) contained a canonical dGTPase domain, or (ii) contained at least two distinct annotated domains, including a dGTPase domain. This filtering aimed to identify both typical dGTPases and potential multifunctional fusion proteins. The resulting tree in Newick format was visualized using the ete3 Python package (version 3.1.3).

### Structure prediction with AlphaFold3

To investigate structural features of the identified dGTPase-related proteins, we employed AlphaFold3 (version 3.0.1) for 3D structure prediction. Unless otherwise specified, all predictions were carried out using default parameters. For potential multimeric proteins, we generated homomeric octamer models as input to the multimer mode of AlphaFold3 to evaluate possible oligomerization states and interface conservation.

### Protein expression and purification

Vdg and its variants were cloned into pRSFDuet-1 expression vectors containing an N-terminal 6×His-SUMO tag with an integrated ubiquitin-like protease 1 (ULP1) cleavage site. Heterologous expression was carried out in *Escherichia coli* BL21(DE3) cells grown at 37 °C until mid-log phase (OD_600_ = 0.6–0.8). Protein expression was induced by reducing the temperature to 20 °C and adding 0.5 mM isopropyl β-D-1-thiogalactopyranoside (IPTG), followed by overnight incubation.

Cells were harvested by low-speed centrifugation (4100 × *g*, 15 min, 4 °C) and lysed by sonication in ice-cold lysis buffer (50 mM Tris-HCl, pH 7.5, 300 mM NaCl, 20 mM imidazole). Lysates were clarified by high-speed centrifugation (30,000 × *g*, 30 min, 4 °C) and applied to a Ni-NTA resin column for immobilized metal affinity chromatography (IMAC). Bound proteins were eluted using a linear imidazole gradient (20–500 mM) in Tris-NaCl buffer (pH 7.5).

The SUMO tag was removed by incubation with ULP1 protease at 4 °C for 2 h in dialysis buffer (50 mM Tris-HCl, pH 8.0, 150 mM NaCl). The cleaved protein was further purified by anion-exchange chromatography on a HiTrap Q HP column using a linear NaCl gradient (0.15–1.0 M) in 50 mM Tris-HCl (pH 8.0). After verification by analytical SDS-PAGE, the protein was concentrated and subjected to Superdex 200 Increase 10/300 GL size-exclusion chromatography column (GE Healthcare) equilibrated with 20 mM HEPES (pH 7.5) and 300 mM NaCl. Monodisperse peak fractions were concentrated to 5 mg/mL, flash-frozen in liquid nitrogen, and stored at −80 °C.

### Sample preparation for structural analysis

For the Vdg-dGTP-dAMP complex, the purified H114A mutant was concentrated to 5 mg/mL and incubated with 5 mM small molecules for 2 h at 4 °C before grid preparation. For cryo-EM sample deposition, 3 μL aliquots of either the ligand-free protein or the ligand-bound complex were applied to glow-discharged Quantifoil Au R1.2/1.3, 300-mesh grids. Vitrification was performed using a plunge-freezing device under cryo-optimized conditions (4 °C, 100% humidity), with a blotting time of 5 s, followed by rapid immersion in liquid ethane for cryopreservation.

### Electron microscopy data acquisition

High-resolution cryo-EM data were collected on a Titan Krios transmission electron microscope (Thermo Fisher Scientific) operating at 300 kV, equipped with a BioQuantum GIF/K2 Summit direct electron detector (Gatan). Automated data acquisition was performed in super-resolution mode at a nominal magnification of 130,000×, with a 20 eV energy filter slit width applied during acquisition. Dose-fractionated exposures utilized a total accumulated dose of 60 e⁻/Å², delivered at a flux rate of 9.2 e⁻/Å²/s through a 32-frame video acquisition protocol. The optical system was configured with intentional defocus values ranging from 1.2 to 1.8 μm, while detector calibration established a super-resolved pixel dimension of 0.52 Å per pixel. Beam-image shift strategies^46^ were systematically employed for multi-position data collection across grid regions.

### Cryo-EM data processing

For the apo Vdg and Vdg-ligands complex datasets, a total of 2547 and 6896 super-resolution micrographs, respectively, were subjected to motion correction, dose-weighting, and frame integration using MotionCor2^47^. The corrected micrographs were subsequently imported into cryoSPARC (v4.2.1)^48^ for downstream processing. Detailed processing workflows, including particle picking, 2D classification, and 3D refinement, are provided in Supplementary Figs. 2 and 4.

### Model building and refinement

For the atomic model of apo Vdg, the structure of Vdg predicted by AlphaFold^49^, as the initial model, was manually fitted in UCSF Chimera^50^ and checked in COOT^51^. The corrected model was further refined by real-space refinement in PHENIX^52^. CIF files for ligands were generated in PHENIX using eLBOW^53^. In COOT and PHENIX, with the apo Vdg as the initial model, the atomic model of ligands-bound Vdg was generated by several rounds of real-space refinement. dGTP and dAMP were fitted into the density using COOT. The resulting model was then manually rebuilt in COOT and further refined by real-space refinement in PHENIX. The final refinement statistics are provided in Supplementary Table 1. All figures were prepared with UCSF ChimeraX^54^ or Pymol (PyMOL Molecular Graphics SYtem, v.2.3.4, Scöhrödinger) (https://pymol.org/2/).

### dGTPase activity assays

dGTPase activity was measured by incubating Vdg or its mutants with dGTP in reaction buffer containing 100 mM Tris-HCl (pH 8.0), 2 mM MgCl_2_, and 10 μM MnCl_2_. To target the dAMP allosteric pocket, dAMP was added in a molar ratio of 1:1 relative to the protein. Reactions were initiated by adding the enzyme to the reaction mixture containing the substrates, followed by incubation at room temperature for 30 min. Control reactions were prepared identically but without the addition of Vdg protein. All reactions were quenched by heating at 95 °C for 10 min, followed by centrifugation at 13,000 × *g* for 10 min to remove precipitated proteins. The supernatants were then applied to a HiTrap Q HP anion-exchange column and eluted using 2 M ammonium acetate. Elution profiles were monitored by measuring absorbance at 260 nm, generating nucleotide hydrolysis curves corresponding to the enzymatic activity. To assess protein stability during reaction, samples were taken at the start of the reaction and 1 h after the reaction began. The samples were centrifuged at 13,000 × *g* for 10 min, and the supernatants were collected. The protein content in the supernatants was then evaluated by SDS-PAGE analysis.

### Size exclusion chromatography coupled to multi-angle light scattering (SEC-MALS)

The sample was prepared at 0.9 mg/mL in buffer (20 mM Tris-HCl, pH 7.5, 300 mM NaCl, 1 mM DTT), and 50 μL was subjected to SEC-MALS analysis on a BioCore SEC-500 column (NanoChrom, 0.5 mL/min flow rate). Light scattering and refractive index data were collected using a DAWN HELEOS detector and an Optilab rEX detector (Wyatt Technology), respectively. Molecular weight was subsequently calculated with ASTRA software (Wyatt Technology).

### Phage cultivation and plaque assays

Bacteriophages T2 (ATCC 11303-B2), T4 (ATCC 11303-B4), T5 (ATCC 11303-B5), T6 (ATCC 11303-B6), T7 (ATCC BAA-1025-B2), and ΦX174 (ATCC 13706-B1). *E. coli* MG1655 was cultured in LB broth at 37 °C until reaching an OD_600_ of 0.8–1.0. A 500 μL aliquot of this culture was then mixed with molten top agar (0.8% agar in LB broth) and poured onto a 15 cm LB agar plate to solidify, forming the bottom agar layer. Freeze-dried phage stocks from ATCC were reconstituted by adding 500 μL of fresh LB broth to each vial. Subsequently, 5 μL drops of the reconstituted phage suspension were spotted onto the surface of the prepared double-layer agar plates. Following overnight incubation at 37 °C, phage plaques were harvested by scraping into LB broth. The resulting suspension was centrifuged at 2400 × *g* for 25 min to pellet cellular debris. The supernatant was filtered through a 0.22 μm membrane filter (Millipore). For phage amplification, 2 mL of the filtered supernatant was used to infect an *E. coli* MG1655 culture grown in LB broth to an OD_600_ of 0.5–0.8. The infected culture was incubated overnight at 37 °C with shaking (220 rpm) until complete lysis was observed (culture clearing). The lysate was centrifuged, and the supernatant was filter-sterilized through a 0.22 μm membrane to obtain the purified phage stock solution.

Phage titer was determined using the small drop plaque assay method^55^. Briefly, the reconstructed plasmid was transformed into chemically competent *E. coli* BL21(DE3) cells. A single transformed colony was picked from a fresh LB agar plate (containing appropriate antibiotic) and grown in LB broth with antibiotic at 37 °C to an OD_600_ of ~0.4. A 1 mL aliquot of this *E. coli* BL21(DE3) culture was mixed with 6 mL of molten top agar containing 50 μg/mL Kanamycin and 0.1 mM IPTG. This mixture was immediately poured onto a 15 cm LB agar plate and incubated at 37 °C for 30 min to allow the top agar to solidify and pre-induce expression. The phage stock solution was serially diluted 10-fold in LB broth. Then, 2 μL drops of each dilution were spotted onto the surface of the prepared double-layer agar plates. After the drops were air-dried, the plates were inverted and incubated overnight at 37 °C. Plaques were counted and photographed.

### Reporting summary

Further information on research design is available in the Nature Portfolio Reporting Summary linked to this article.

## Supplementary information

Supplementary Information

Reporting Summary

Transparent Peer Review file

## Source data

Source Data

## Data Availability

The cryo-EM density maps have been deposited into the Electron Microscopy Data Bank under accession numbers EMD-65392 (apo), EMD-65393 (H114A with dGTP and dAMP), and EMD-65396 (Two octameric assemblies H114A with dGTP and dAMP). The atomic coordinates have been deposited at the Protein Data Bank under accession numbers 9VWA (apo), 9VWC [(VWC/pdb)] (H114A with dGTP and dAMP), and 9VWG (Two octameric assemblies H114A with dGTP and dAMP). A Source data file is included with this manuscript. Source data are provided with this paper.
