# Supplementary material for: Octameric dGTPase assemblies mediate broad anti-phage defense
Source: Nat Commun. 2026 Jul 25;17:9103. doi: 10.1038/s41467-026-75805-z (PMC13519002; doi:10.1038/s41467-026-75805-z)
Supplement: Supplementary file 1 [file 41467_2026_75805_MOESM1_ESM.pdf]

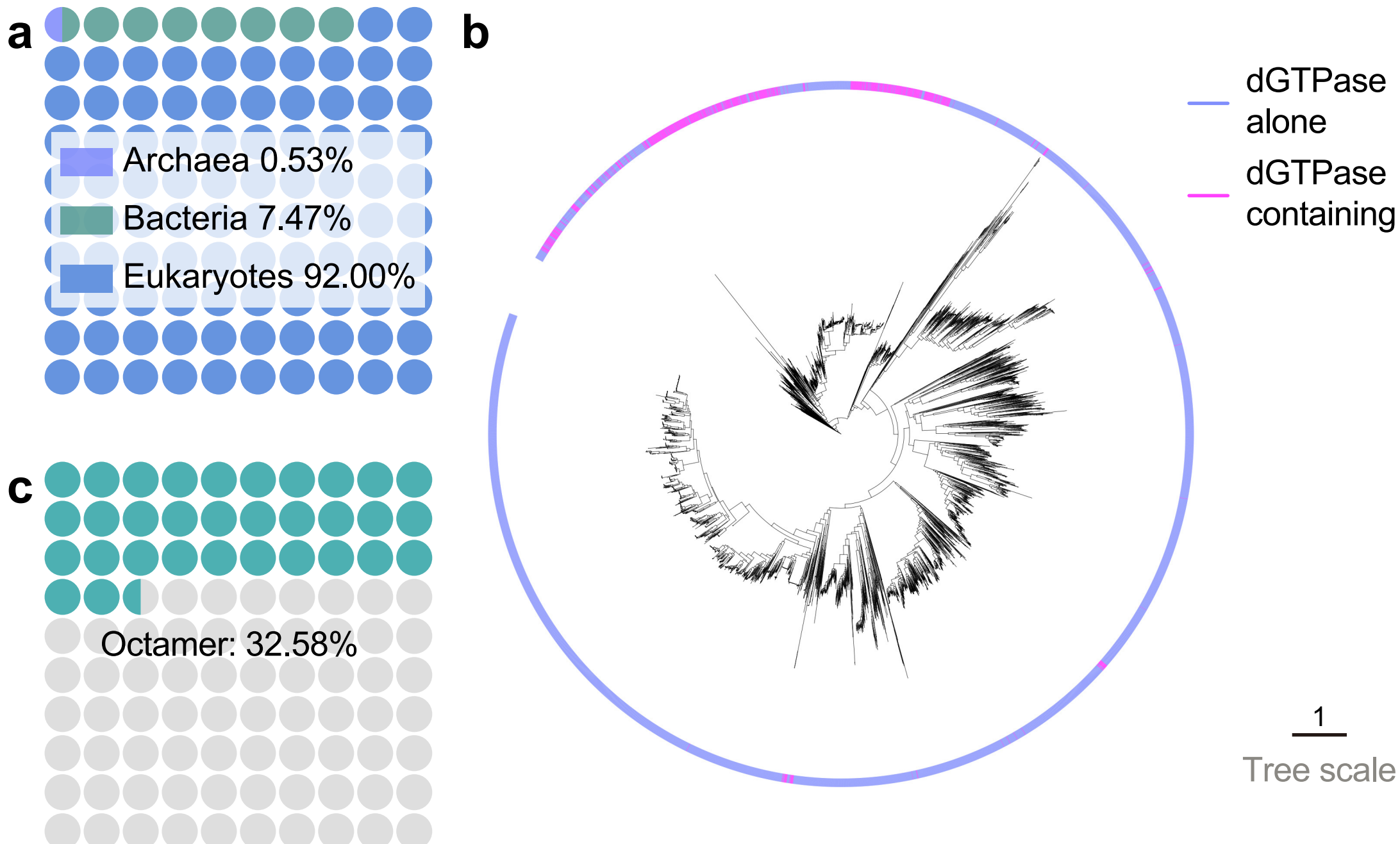

Supplementary Fig. 1 | Distribution and evolutionary analysis of dGTPase-containing proteins across archaea, bacteria, and eukaryotes.
a. Percentage of proteins containing the dGTPase module in the indicated three domains of life.
b. Phylogenetic tree based on dGTPase domain sequences, constructed using the dGTPase domain alone. Proteins containing only the dGTPase domain are highlighted in purple, while those containing dGTPase fused to other domains are shown in magenta.
c. Distribution of AlphaFold-predicted dGTPase structures with an iPTM above 0.6, representing high-confidence octameric candidates (out of 792 total proteins screened).

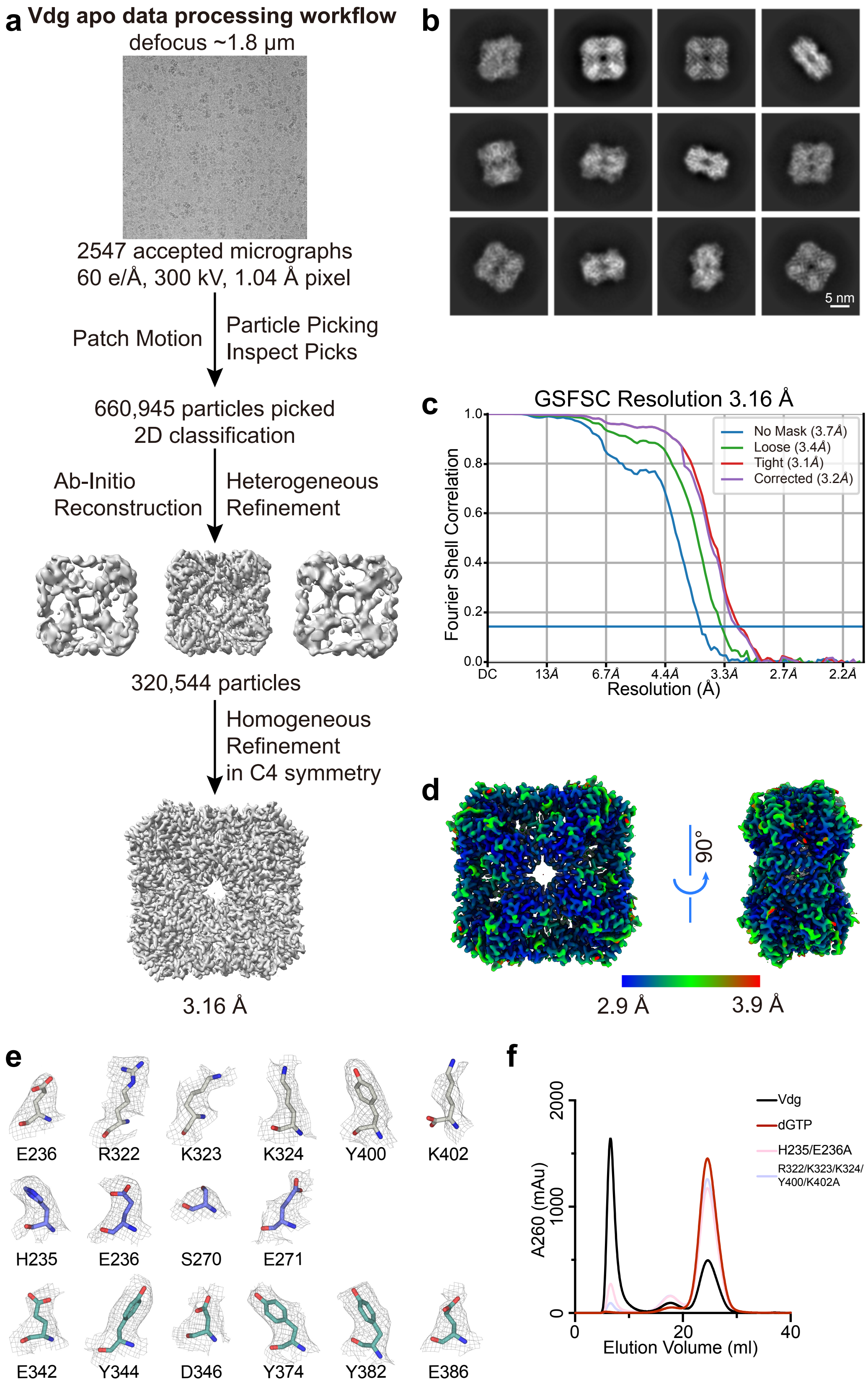

Supplementary Fig. 2 | Cryo-EM data processing workflow of Vdg octamer.
a. Data processing flowchart of the Vdg in the apo state.
b. Representative 2D class averages of apo Vdg particles.
c. Map resolution estimated by the gold-standard Fourier shell correlation (GSFSC) with a cutoff at 0.143.
d. Local resolution distribution of Vdg apo structure.
e. Cryo-EM density map (contoured at 8.5 σ) showing side chains of key residues involved in the dimer-to-octamer interface.
f. *In vitro* chromatographic assays comparing wild-type Vdg with interface mutants, showing dGTP hydrolysis activity.
Data are representative of at least three independent experiments.

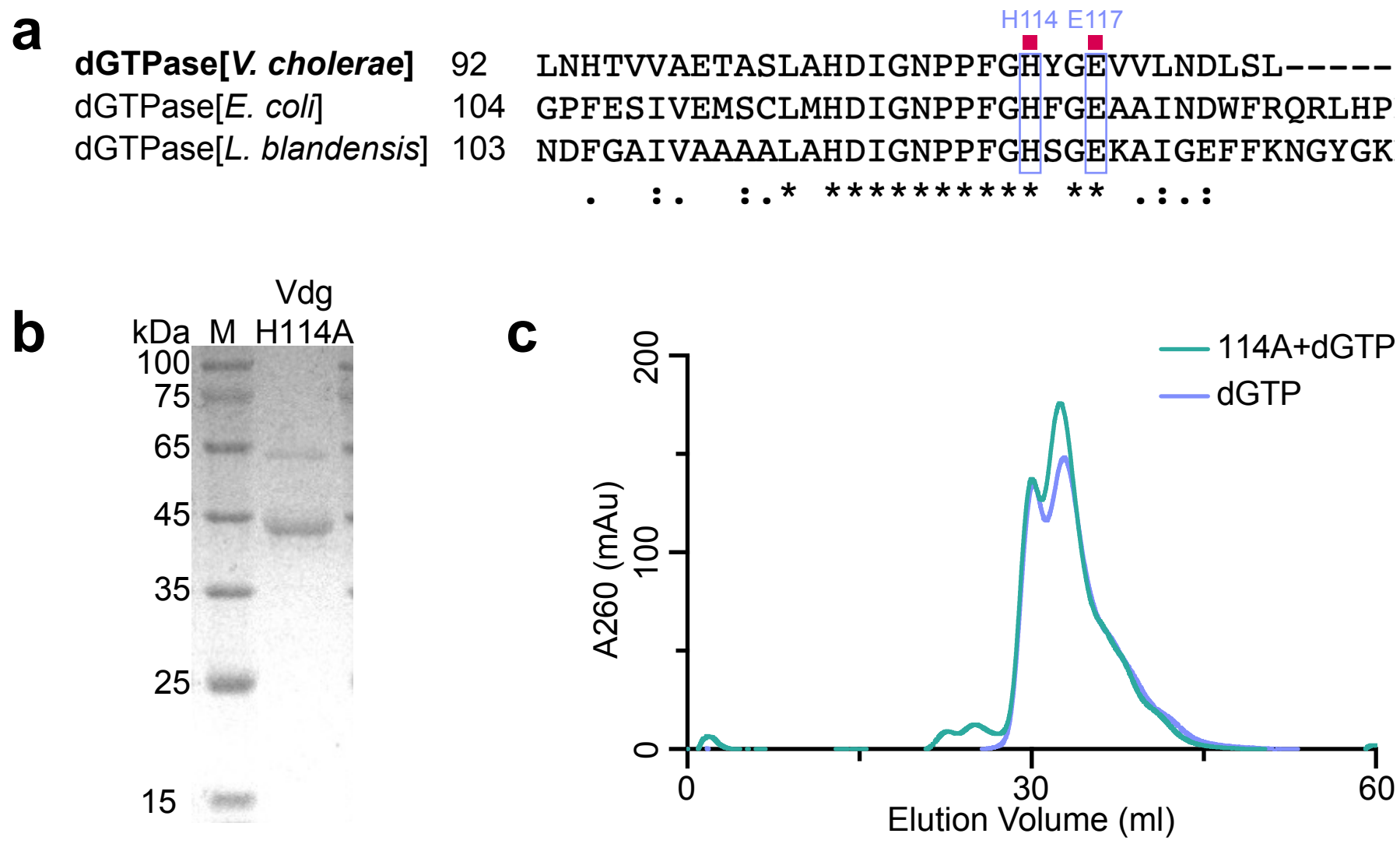

Supplementary Fig. 3 | Active site of Vdg dGTPase.

a. Multiple sequence alignment of selected dGTPase homologs, focusing on the region containing residues critical for catalytic activity. His114 and Glu117 of Vdg are highlighted in the purple box. The sequences included in the alignment are as follows: *Vibrio cholerae* serotype O1 (Uniprot ID Q9KMM3); *Escherichia coli* dGTPase (P15723); *Leeuwenhoekiella blandensis* dGTPase (A3XHN1).

b. SDS-PAGE analysis confirming the purity of the Vdg H114A mutant protein.

c. dGTP hydrolysis assay demonstrates that the Vdg H114A mutant abolishes dGTPase activity.

Data are representative of at least three independent experiments.

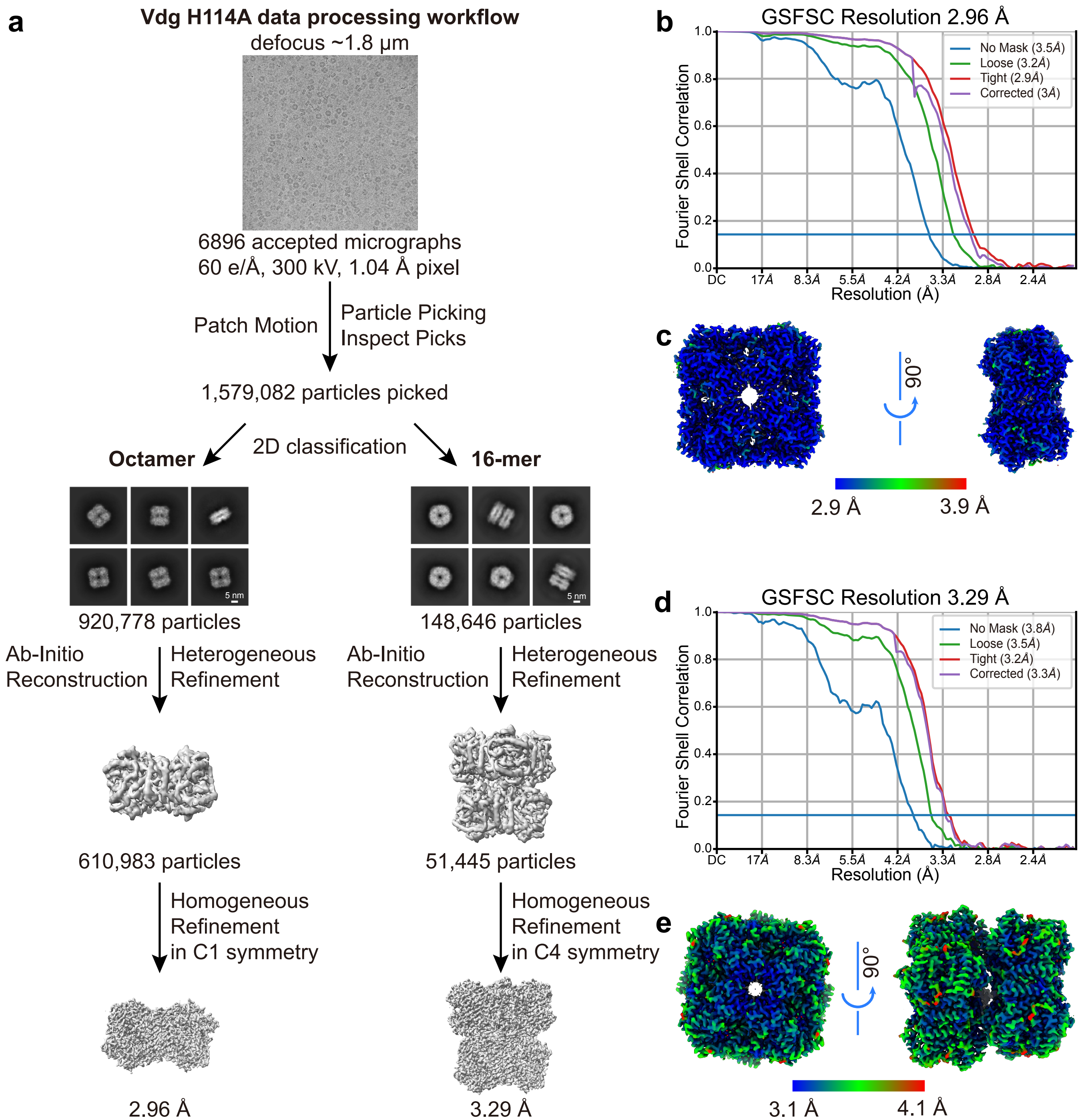

Supplementary Fig. 4 | Cryo-EM data analysis of Vdg H114A mutant bound to dGTP and dAMP.
a. Data processing workflow for Vdg H114A mutant complexed with dGTP and dAMP.
b. Map resolution of octameric Vdg H114A with ligands estimated by the gold-standard Fourier shell correlation (GSFSC) with a cutoff at 0.143.
c. Local resolution distribution of octameric Vdg H114A with dGTP and dAMP ligands.
d. Map resolution of 16-mer Vdg H114A with ligands estimated by the gold-standard Fourier shell correlation (GSFSC) with a cutoff at 0.143.
e. Local resolution distribution of 16-mer Vdg H114A with dGTP and dAMP ligands.

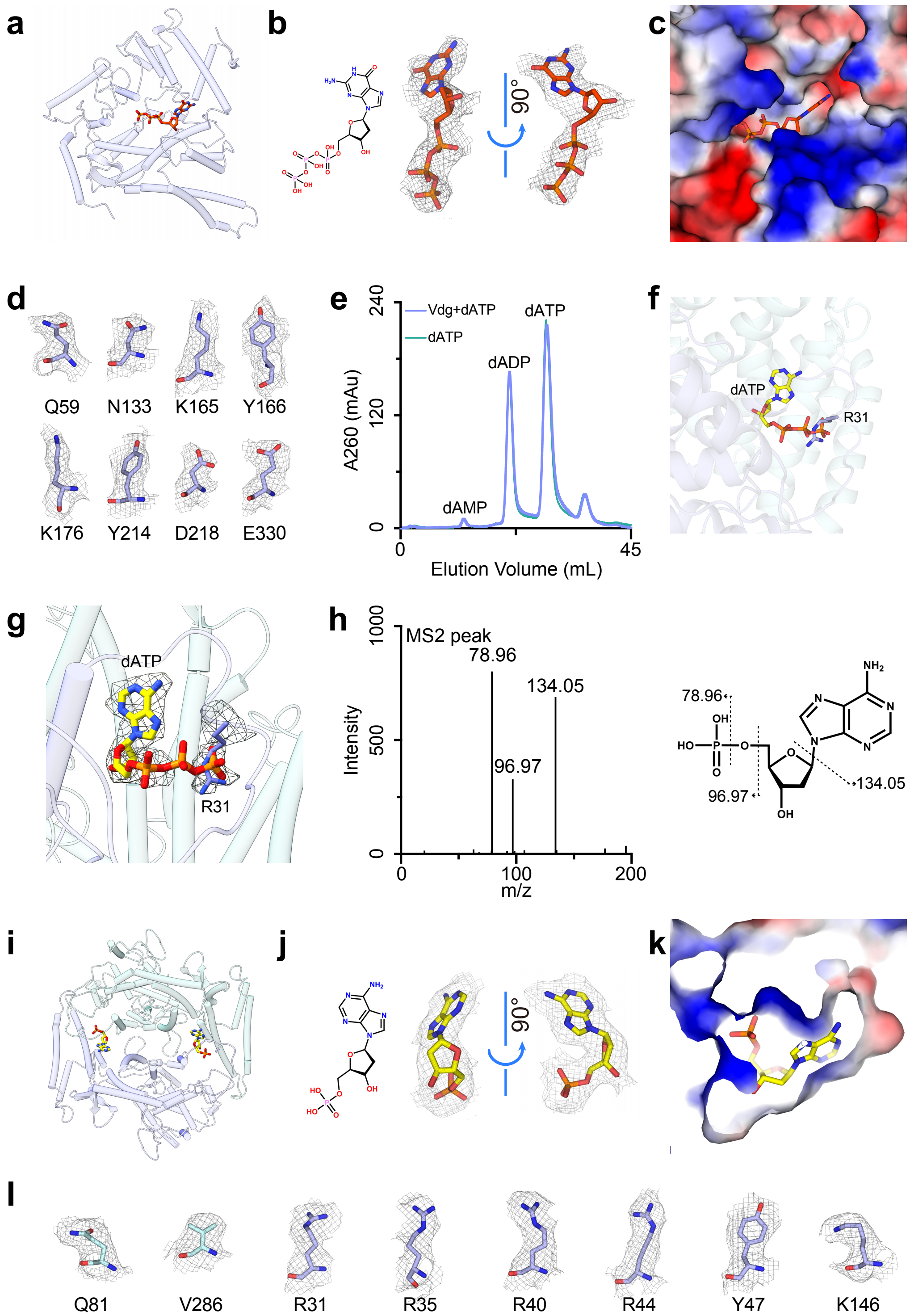

Supplementary Fig. 5 | Structural and biochemical analyses of Vdg hydrolysis activity.
a. The dGTP molecule binds within the catalytic pocket of a Vdg monomer.
b. Chemical structure and corresponding cryo-EM densities of dGTP contoured at 8.5 σ.
c. Electrostatic surface representation of a Vdg monomer highlighting the dGTP binding pocket.
d. Electron densities for key residues involved in the dGTPase catalytic pocket. Cryo-EM density maps contoured at 8.5 σ.
e. Enzymatic assay showing that Vdg does not hydrolyze dATP.
f. Cryo-EM structural modeling reveals a steric clash between dATP and residue R31 in the allosteric pocket, preventing proper binding.
g. Cryo-EM density at the allosteric site is inconsistent with the size and shape of dATP or dADP, indicating these molecules are not bound.
h. MS2 fragmentation spectrum of the dAMP, annotated according to expected fragments of dAMP.
i. Two dAMP molecules are bound at the allosteric sites located at the dimer interface of Vdg H114A.
j. Chemical structure and cryo-EM density of dAMP bound in the allosteric pocket, contoured at 8.5 σ.
k. Electrostatic surface representation of two Vdg subunits, highlighting the allosteric dAMP binding pocket.
l. Cryo-EM density map for key residues involved in the allosteric pocket, contoured at 8.5 σ.

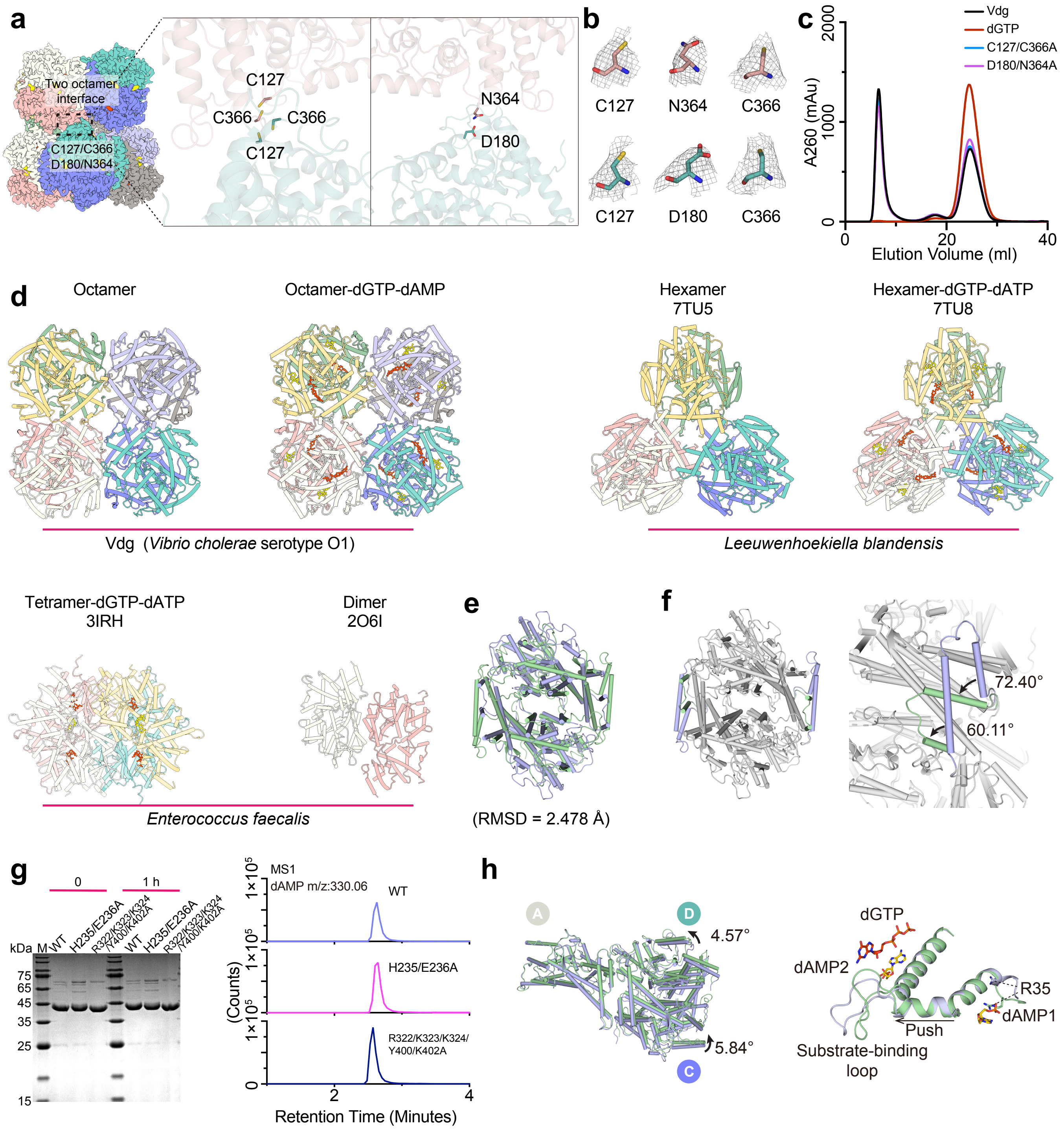

Supplementary Fig. 6 | Structure analysis of the octamer-octamer interface in Vdg.

a. Schematic representation showing the octamer-octamer assembly in the Vdg 16-mer. The catalytic site is indicated by a dashed rectangle (left). Detailed view (right) highlights key residues involved in interactions, shown as sticks.

b. Cryo-EM density maps of key residues contoured at 3 σ.

c. *In vitro* chromatography assays assessing dGTP hydrolysis by wild-type Vdg and interface mutants. Data are representative of at least three experiments.

d. Schematic representations of octameric, hexameric, tetrameric, and dimeric dGTPases, in the presence or absence of bound small molecules, from *Vibrio cholerae* serotype O1, *Leeuwenhoekiella blandensis*, and *Enterococcus faecalis*. dGTP and dAMP/dATP are colored red and yellow, respectively.

e. Structural alignment of dimeric units from *Vibrio cholerae* serotype O1 (colored in purple) and *Leeuwenhoekiella blandensis* (colored in green).

f. Highlighted structural differences between dimeric units from octameric and hexameric conformations. In the octamer, two helices (G226-R266; purple) differ markedly from their counterparts in the hexamer (G269-L296; green), exhibiting angular displacements of 72.40° and 60.11°, respectively.

g. Left: SDS-PAGE analysis confirming the purity and stability of Vdg protein and its mutants before and after the enzymatic reaction.
Right: Mass spectrometry analysis confirming dAMP binding to Vdg protein and its mutants.

h. Left: Structural alignment of apo Vdg (purple) and Vdg in complex with dGTP and dAMP (green), relative to chain A. Showing angular displacements of chain C (5.84°) and chain D (4.57°).
Right: A proposed activation model in which dAMP binding at the allosteric site induces conformational changes that push the helix and reposition the substrate-binding loop, thereby promoting dGTPase activity. dAMP is shown in yellow and dGTP in red.

Supplementary Table 1 | Cryo-EM data collection, refinement and validation statistics

	Vdg apo (EMD-65392) (PDB 9VWA)	Octamer H114A with dGTP and dAMP (EMD-65393) (PDB 9VWC)	16-mer H114A with dGTP and dAMP (EMD-65396) (PDB 9VWG)
**Data collection and processing**			
Magnification	130,000	130,000	130,000
Voltage (kV)	300	300	300
Electron exposure (e–/Å^2)	60	60	60
Defocus range (μm)	-1.2 ~ -1.8	-1.2 ~ -1.8	-1.2 ~ -1.8
Pixel size (Å)	1.04	1.04	1.04
Symmetry imposed	C4	C1	C4
Initial particle images (no.)	660,945	920,778	148,646
Final particle images (no.)	320,544	610,983	51,445
Map resolution (Å)	3.16	2.96	3.29
FSC threshold	0.143	0.143	0.143
Map resolution range (Å)	-	-	-
**Refinement**			
Initial model used (PDB code)	AF3	AF3	AF3
Model resolution (Å)	3.4	3.1	3.4
FSC threshold	0.5	0.5	0.5
Model resolution range (Å)	-	-	-
Map sharpening *B* factor (Å)	153.5	137.0	123.9
Model composition			
Non-hydrogen atoms	25,832	26,216	52,432
Protein residues	3,216	3,216	6,432
Ligands	0	16	32
B factor (Å^2)			
Protein	31.89	39.95	32.40
Ligands	-	43.30	36.84
R.m.s. deviations			
Bond lengths (Å)	0.003	0.004	0.004
Bond angles (°)	0.430	0.907	0.910
Validation			
MolProbity score	1.58	1.58	1.30
Clashscore	5.04	4.93	5.61
Poor rotamers (%)	0.00	0.00	0.00
Ramachandran plot			
Favored (%)	95.47	95.44	98.31
Allowed (%)	4.53	4.56	1.69
Disallowed (%)	0	0	0
