# Supplementary material for: Octameric dGTPase assemblies mediate broad anti-phage defense
Source: Nat Commun. 2026 Jul 25;17:9103. doi: 10.1038/s41467-026-75805-z (PMC13519002; doi:10.1038/s41467-026-75805-z)
Supplement: Supplementary file 3 [file 41467_2026_75805_MOESM3_ESM.pdf]

nature portfolio

Peer Review File

Octameric dGTPase Assemblies Mediate Broad Anti-phage Defense

Corresponding Author: Professor Ang Gao

This file contains all reviewer reports in order by version, followed by all author rebuttals in order by version.

Version 0:

Reviewer comments:

Reviewer #1

(Remarks to the Author)
Shi et al. present structural and functional analyses of a dGTPase involved in anti-phage defense. Comparative genomics indicates that dGTPases are broadly distributed across the tree of life, underscoring their potential importance. The authors determine cryo-EM structures of a Vibrio dGTPase that assembles as an octamer and as filaments. Functional assays show comparable catalytic activity for the octamer and filaments, suggesting the octamer is the operative unit. Additionally, the authors identify dAMP as an allosteric ligand of dGTPase. The work is exciting and the conclusions are generally well supported by the data. A few minor points, if addressed, would further improve clarity.

Minor concerns:
1. In lines 171-174, it appears the oligomeric state of homologs was inferred bioinformatically rather than biochemically. Please clarify the specific sequence/structural features or models used to predict octameric assemblies, and state the confidence/limitations of these predictions.

2. Supplemental Fig. 2F does not clearly demonstrate that the dimer is inactive while the octamer is active. Please soften the claim or provide direct enzymatic comparisons between dimeric and octameric species in line 204.

3. Please explain why dGTPase preferentially binds dAMP rather than dADP or dATP. In addition, please comment on whether non-hydrolyzable dATP analogs trigger the same conformational changes observed with dAMP.

4. The eight subunits are difficult to distinguish in Figure 3. Consider revising the color palette to better convey the octameric architecture.

Reviewer #2

(Remarks to the Author)
I co-reviewed this manuscript with one of the reviewers who provided the listed reports. This is part of the Nature Communications initiative to facilitate training in peer review and to provide appropriate recognition for Early Career Researchers who co-review manuscripts.

Reviewer #3

(Remarks to the Author)
Shi et.al. conducted bioinformatics studies and identified ~ 1,000 dGTPases from all three kingdoms of life. They then did structural mining and identified dGTPases with potential octameric organization, a previously unknown configuration. Eventually, the authors selected a dGTPase from Vibrio cholerae (Vdg) for structural and biochemical studies. Ion-exchange chromatography demonstrated that Vdg converted dGTP to dG, consistent with the predicted dGTPase activity. Cryo-EM investigation further revealed that VdG adopted a tetramer of dimer organization, forming a strikingly symmetric square-shaped, two-layer, octamer. The authors also determined substrate-bound Vdg structure using an active site mutant. Analysis of the substrate-bound Vdg structure surprisingly revealed dAMP at the dimer interface, likely functioning as an

allosteric regulator. Cryo-EM analysis also revealed 16-mer formed by two octamers, and potential filament-like polymers. Vdg displayed broad spectrum anti-phage activity in phage plaque assays. Mutation at the dGTP binding sites, the dAMP binding site, and octameric interface all impaired the anti-phage activity of Vdg, while the interface between octamers was dispensable.

Overall the results are clean and supportive of the conclusions. There are a few points that can improve the work.

Major:

1. The authors should include structural comparison between Vdg and other known antiviral/anti-phage dGTPases, including monomer-monomer interaction and interactions between dimers.
2. The authors should include structural comparison between apo Vdg and substrate-bound Vdg, e.g., conformational changes in the dGTP and dAMP binding pockets (e.g., “open” vs “closed”, their potential crosstalk, and changes at the dimer-dimer interface. Given the authors observed 16-mer or even higher-order assemblies in the substrate-bound form but not the apo form, one would expect notable conformational changes.
3. It is intriguing that dAMP is “deeply buried” in a positively charged pocket at the monomer-monomer interface of each Vdg dimer, and Vdg appears to be a constitutive octamer, yet dAMP does not bind Vdg in the absence of the substrate dGTP. How do the authors envision the coordinated conformational changes during dAMP binding and release? Could dAMP binding be a downstream event of dGTP binding/hydrolysis?

Minor issues:

1. Lines 72-74, the sentence “the strategy of depleting intracellular dNTP pools to inhibit viral replication protrudes the convergence of …” is confusing. Did the authors mean “ the strategy … illustrates the convergence of …”?
2. Lines 131-132, “… canonical signaling modules that mediate initiate immune responses …” should be either “… modules that mediate initial … “ or “… modules that initiate …”.
3. Line 142, “these findings underscore the dGTPase function as versatile immune effector, …”. The functions of most dGTPases identified in the bioinformatics screen haven’t been experimentally validated. It is unclear whether they act as immune effectors or not. The authors should consider soften the tone, “these findings suggest that …”.
4. Line 146-147, “In our analysis of dGTPase-related defense systems, we observed that dGTPase frequently replaces proteins known for their aggregation potential”. “replace” is confusing here. Do the dGTPases replace other aggregation-prone proteins at genetic levels? A couple of examples would clarify the issue.
5. Figure 1c, protein standards with known molecular weight should be marked on the SEC profile for proper evaluation of Vdg size in solution; SEC-MALS would be a more definitive way to determine molecular weight/oligomerization state.
6. Figure 1d, dG and dGTP standards should be included.
7. Figure 2a, please label molecules A, B, C and D so the readers can place the close-in views in the context.
8. In Figure 4b, where are H/A114 and E117? Are they close to the bound dGTP? This information helps understand the active site formation.
9. Line 292, are the dimer-to-octamer interfacial mutants H235/E236A and R322/K323/K324/Y400/K402 stable in solution? It can be quickly tested with recombinant proteins. It would be interesting to see whether these mutants are stable and still bind dGTP and/or dAMP.

Version 1:

Reviewer comments:

Reviewer #1

(Remarks to the Author)
The authors have addressed all my concerns. The manuscript is in great shape for publication.

Reviewer #2

(Remarks to the Author)
I co-reviewed this manuscript with one of the reviewers who provided the listed reports. This is part of the Nature Communications initiative to facilitate training in peer review and to provide appropriate recognition for Early Career Researchers who co-review manuscripts.

Reviewer #3

(Remarks to the Author)
The authors have successfully addressed all my concerns.

Open Access This Peer Review File is licensed under a Creative Commons Attribution 4.0 International License, which permits use, sharing, adaptation, distribution and reproduction in any medium or format, as long as you give appropriate credit to the original author(s) and the source, provide a link to the Creative Commons license, and indicate if changes were made.
In cases where reviewers are anonymous, credit should be given to 'Anonymous Referee' and the source.
The images or other third party material in this Peer Review File are included in the article's Creative Commons license, unless indicated otherwise in a credit line to the material. If material is not included in the article's Creative Commons license and your intended use is not permitted by statutory regulation or exceeds the permitted use, you will need to obtain permission directly from the copyright holder.
To view a copy of this license, visit https://creativecommons.org/licenses/by/4.0/

We thank the reviewers for their valuable comments. In the revised manuscript, we have performed more experiments and have carefully addressed the reviewers' concerns. Changes in the manuscript are highlighted with underlined text. We hope that these revisions improve the overall quality and clarity of the manuscript. Our detailed responses to reviewers' critiques are provided below (reviewers' comments are in red, and our responses are in black).

REVIEWER COMMENTS

****

Reviewer #1 (Remarks to the Author):

Shi et al. present structural and functional analyses of a dGTPase involved in anti-phage defense. Comparative genomics indicates that dGTPases are broadly distributed across the tree of life, underscoring their potential importance. The authors determine cryo-EM structures of a Vibrio dGTPase that assembles as an octamer and as filaments. Functional assays show comparable catalytic activity for the octamer and filaments, suggesting the octamer is the operative unit. Additionally, the authors identify dAMP as an allosteric ligand of dGTPase. The work is exciting and the conclusions are generally well supported by the data. A few minor points, if addressed, would further improve clarity.

We thank the reviewer for summarizing the key contributions of our work and finding this study "exciting and the conclusions are generally well supported by the data". By following the reviewer's suggestions, we have performed additional experiments and made necessary revisions to address the reviewer's concerns.

Minor concerns:

1. In lines 171-174, it appears the oligomeric state of homologs was inferred bioinformatically rather than biochemically. Please clarify the specific sequence/structural features or models used to predict octameric assemblies, and state the confidence/limitations of these predictions.

We thank the reviewer for pointing out this issue. In the original manuscript (lines 171-174), we appeared to infer the oligomeric states of homologs bioinformatically rather than determine them biochemically. We have now corrected this description in the main text (line 176).

We clarified the application of the AlphaFold 3 AI model for predicting octameric assemblies. Specifically, we used the Foldseek server to perform structural similarity searches based on octameric 3D features. Subsequently, the AlphaFold 3 AI model was used to predict the octameric state of dGTPases and to evaluate the quality of the interfaces between subunits (lines 175-176).

In addition, we experimentally validated a subset of the predictions and identified an octameric assembly similar to that of *Pseudomonas aeruginosa* PAO1 dGTPase. This result supports the reliability of our structural prediction pipeline. However, we cannot conclude that all dGTPases with iPTM > 0.6 form octameric assemblies, as not all high-scoring candidates were experimentally tested. We have added a discussion of the confidence and limitations of this bioinformatic screening approach. We updated the corresponding text in lines 332-337 to reflect these changes.

2. Supplemental Fig. 2F does not clearly demonstrate that the dimer is inactive while the octamer is active. Please soften the claim or provide direct enzymatic comparisons between dimeric and octameric species in line 204.

We thank the reviewer for this helpful suggestion. We agree that Supplementary Fig. 2f does not allow a direct enzymatic comparison between isolated dimeric and octameric species. Our intention was to indicate that the extensive inter-chain interactions connecting the four dimers within the octamer are important for enzymatic activity, as mutations disrupting these interfaces reduced activity. To avoid overinterpretation, we have revised the sentence in the main text as follows: “These extensive inter-chain interactions, which connect the four dimers within the octamer, are important for enzymatic activity” (lines 208-209).

3. Please explain why dGTPase preferentially binds dAMP rather than dADP or dATP. In addition, please comment on whether non-hydrolyzable dATP analogs trigger the same conformational changes observed with dAMP.

We thank the reviewer for raising this important point. The structural features of the allosteric pocket provide an explanation for the preferential binding of dAMP. Specifically, while the pocket can readily accommodate dAMP, dADP or dATP would introduce additional phosphate groups that are incompatible with the observed pocket geometry and would cause steric clashes with surrounding residues, particularly R31 (Supplementary Fig. 5f,g).

Non-hydrolyzable dATP analogs would still retain these additional phosphate groups and are therefore unlikely to fit into the pocket because of the same steric constraints. In addition, because they cannot be converted into dAMP, they would not be expected to induce the conformational changes associated with dAMP binding.

These points have now been clarified in the revised manuscript (lines 241-247): "Structurally, this pocket is compatible with dAMP, whereas dADP or dATP would introduce additional phosphate groups that are incompatible with the observed pocket geometry and would cause steric clashes with surrounding residues, particularly R31 (Supplementary Fig. 5f, g). Accordingly, non-hydrolyzable dATP analogs, which retain these additional phosphate groups and cannot be converted into dAMP, would not be expected to induce the conformational changes associated with dAMP binding."

4. The eight subunits are difficult to distinguish in Figure 3. Consider revising the color palette to better convey the octameric architecture.

We thank the reviewer for this insightful suggestion. Following the reviewer's recommendation, we have revised the color palette to better distinguish the eight subunits and to more clearly convey the octameric architecture in Fig. 3, 4a, 5a, and Supplementary Fig. 6a and b.

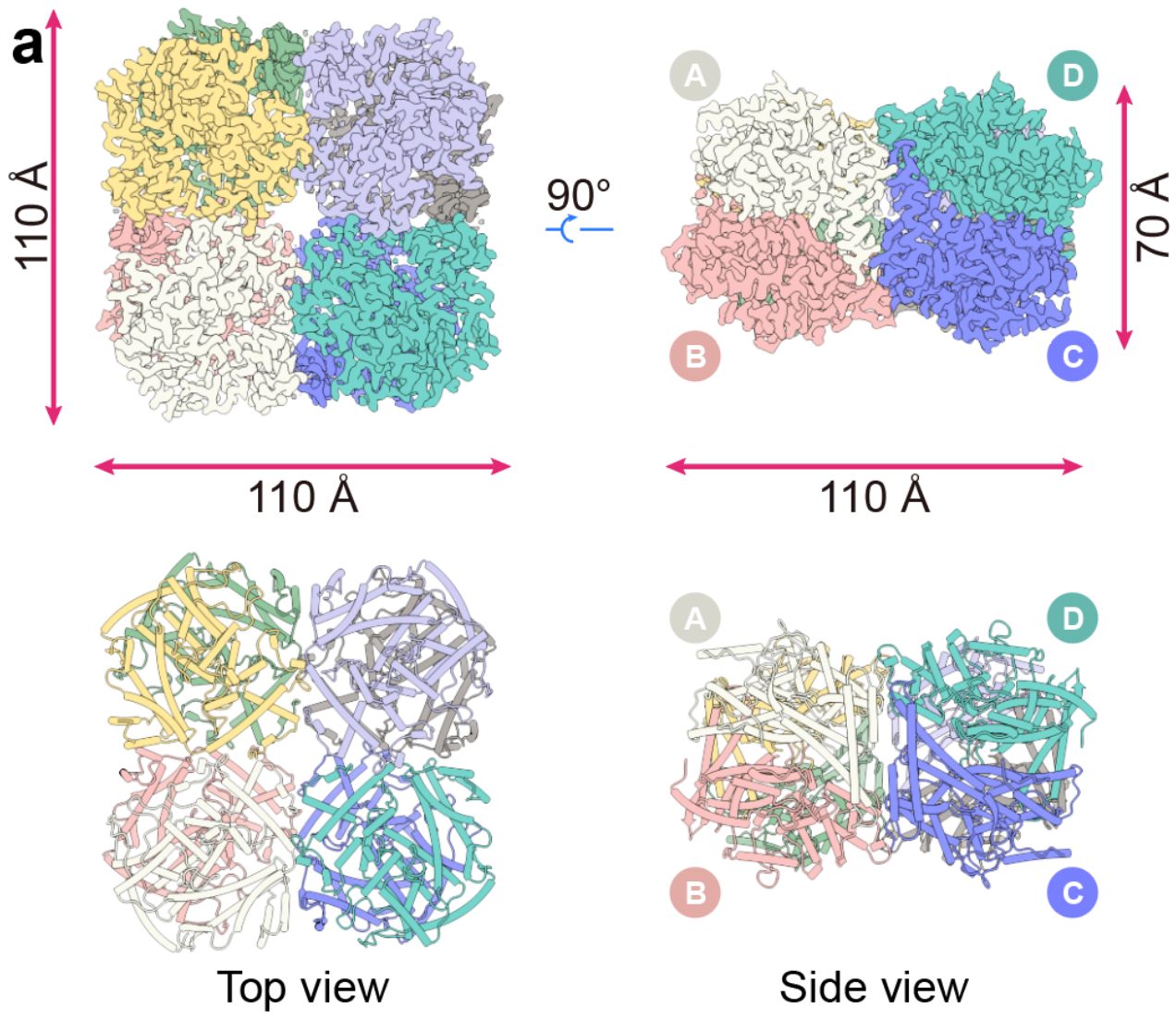

****

Reviewer #2 (Remarks to the Author):

I co-reviewed this manuscript with one of the reviewers who provided the listed reports. This is part of the Nature Communications initiative to facilitate training in peer review and to provide appropriate recognition for Early Career Researchers who co-review manuscripts.

We thank the co-reviewer for their thoughtful evaluation of our manuscript.

****

Reviewer #3 (Remarks to the Author):

Shi et.al. conducted bioinformatics studies and identified ~ 1,000 dGTPases from all three kingdoms of life. They then did structural mining and identified dGTPases with potential octameric organization, a previously unknown configuration. Eventually, the authors selected a dGTPase from Vibrio cholerae (Vdg) for structural and biochemical studies. Ion-exchange chromatography demonstrated that Vdg converted dGTP to dG, consistent with the predicted dGTPase activity. Cryo-EM investigation further revealed that VdG adopted a tetramer of dimer organization, forming a strikingly symmetric square-shaped, two-layer, octamer. The authors also determined substrate-bound Vdg structure using an active site mutant. Analysis of the substrate-bound Vdg

structure surprisingly revealed dAMP at the dimer interface, likely functioning as an allosteric regulator. Cryo-EM analysis also revealed 16-mer formed by two octamers, and potential filament-like polymers. Vdg displayed broad spectrum anti-phage activity in phage plaque assays. Mutation at the dGTP binding sites, the dAMP binding site, and octameric interface all impaired the anti-phage activity of Vdg, while the interface between octamers was dispensable.

Overall the results are clean and supportive of the conclusions. There are a few points that can improve the work.

We thank the reviewer for the positive assessment of our work and for the constructive suggestions. In the revised manuscript, we have performed additional experiments and made revisions to address all the reviewer's concerns.

Major:

1. The authors should include structural comparison between Vdg and other known antiviral/anti-phage dGTPases, including monomer-monomer interaction and interactions between dimers.

We thank the reviewer for this helpful suggestion. In response, we included structural comparisons between Vdg and representative hexameric, tetrameric, and dimeric antiviral/anti-phage dGTPases. These analyses show that Vdg is most similar to the hexameric dGTPase architecture at the level of the dimeric building block, with highly similar monomer-monomer interfaces, allosteric sites, and catalytic pockets (Supplementary Fig. 6d). However, the higher-order assemblies differ in how these dimers pack against one another. In Vdg, two helices at the inter-dimer interface adopt markedly different orientations, with angular displacements of 72.4° and 60.11°, respectively, generating additional dimer-dimer contacts in the octamer.

We have incorporated these structural comparisons into the Discussion (lines 341-354 and Supplementary Fig. 6d, e and f): “To place this architecture in context, we compared Vdg with representative dGTPases of different oligomeric states. The Vdg structure contains eight subunits organized as four dimers, with each monomer harboring one catalytic pocket. By comparison, the hexameric dGTPase from *Leeuwenhoekiella blandensis* (PDB: 7TU5 and 7TU6) contains six subunits organized

as three dimers, whereas the dNTPase from *Enterococcus faecalis* has been observed in both dimeric and tetrameric forms (Supplementary Fig. 6d). At the level of the dimeric building block, Vdg is most similar to the hexameric dGTPase: the dimers can be well superimposed (RMSD = 2.478 Å) and share highly similar monomer-monomer interfaces, allosteric sites, and catalytic pockets (Supplementary Fig. 6e). However, the higher-order assemblies differ in how these dimers pack against one another. In Vdg, two helices at the inter-dimer interface adopt markedly different orientations, with angular displacements of 72.4° and 60.11°, respectively, thereby generating additional dimer-dimer contacts that support the octameric assembly (Supplementary Fig. 6f). Together, these observations highlight both the conserved dimeric core and the diversified higher-order architectures of antiviral dGTPases."

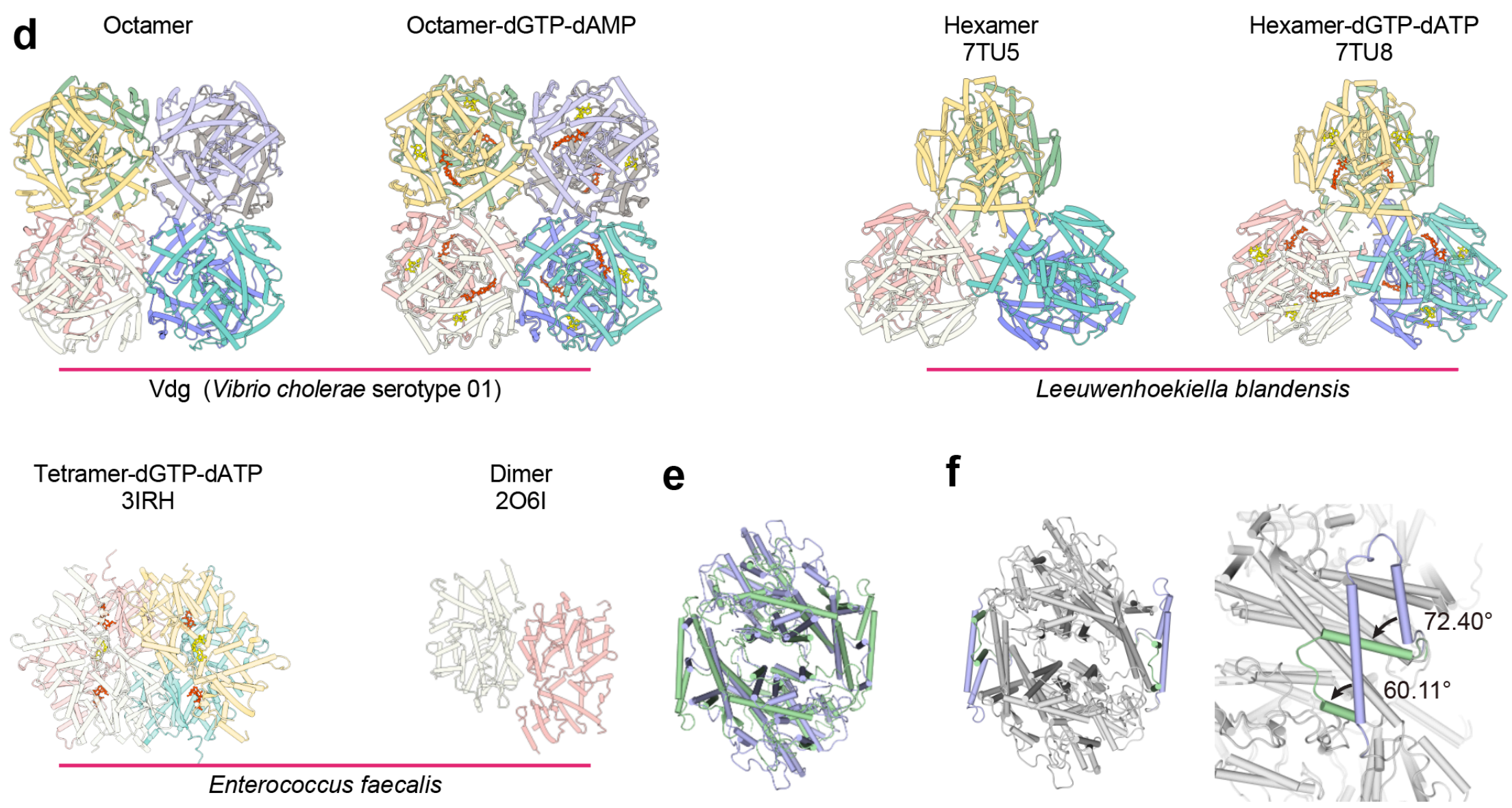

2. The authors should include structural comparison between apo Vdg and substrate-bound Vdg, e.g., conformational changes in the dGTP and dAMP binding pockets (e.g., "open" vs "closed", their potential crosstalk, and changes at the dimer-dimer interface. Given the authors observed 16-mer or even higher-order assemblies in the substrate-bound form but not the apo form, one would expect notable conformational changes.

We thank the reviewer for this valuable suggestion. In response, we performed structural comparisons between apo Vdg and substrate-bound Vdg and incorporated the resulting analyses into the revised manuscript. These comparisons indicate that

dAMP binding induces an overall shift of the dimer-dimer interface. Specifically, the allosteric sites transmit the effect of dAMP binding directly to a critical substrate-binding loop. The structural comparisons and the proposed activation model have been incorporated into the revised manuscript (lines 375-388 and Supplementary Fig. 6h).

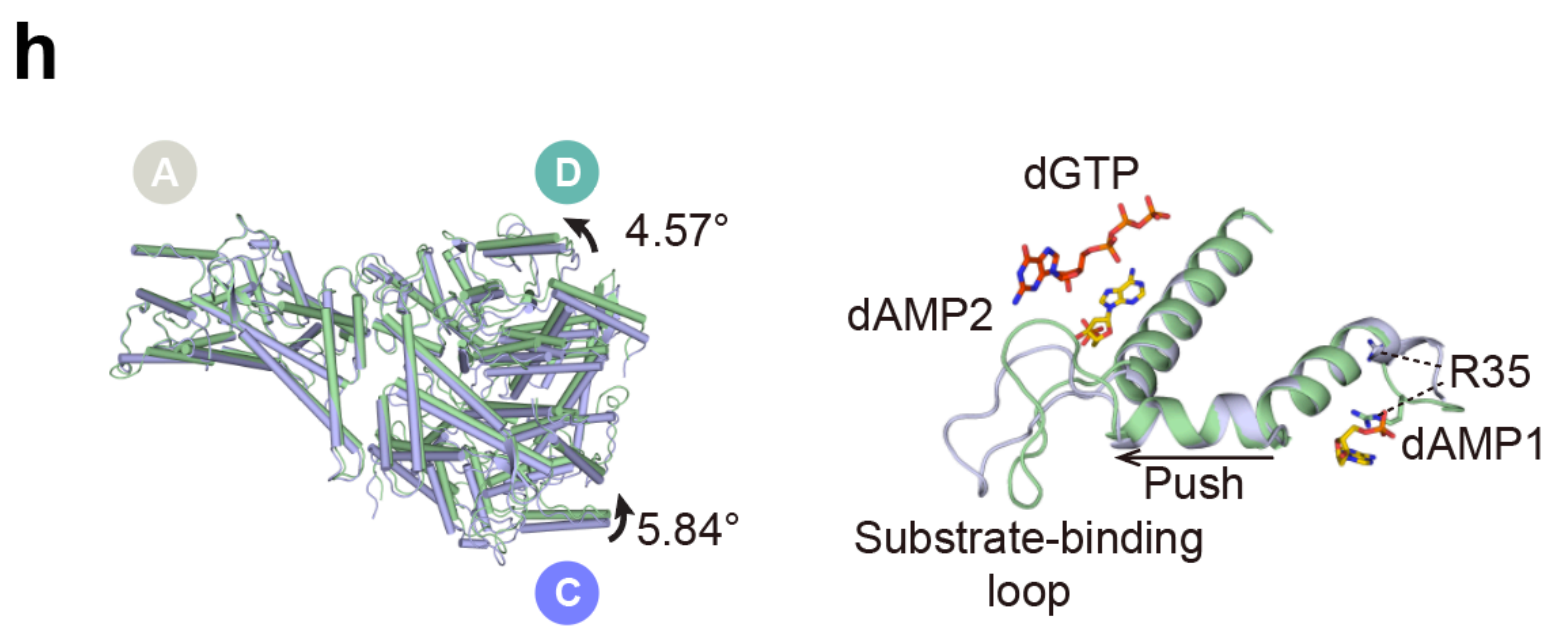

3. It is intriguing that dAMP is "deeply buried" in a positively charged pocket at the monomer-monomer interface of each Vdg dimer, and Vdg appears to be a constitutive octamer, yet dAMP does not bind Vdg in the absence of the substrate dGTP. How do the authors envision the coordinated conformational changes during dAMP binding and release? Could dAMP binding be a downstream event of dGTP binding/hydrolysis?

We appreciate these insightful questions. At present, we do not have direct evidence to define the temporal order of dAMP and dGTP binding or release. Importantly, Vdg retains catalytic activity in the absence of dAMP, indicating that dAMP is not absolutely required for catalysis but rather enhances dGTPase activity when present. Thus, while our current data do not allow us to determine whether dAMP binding is a downstream event of dGTP binding or hydrolysis, they support a model in which dAMP acts as an allosteric enhancer rather than an essential prerequisite for enzymatic activity. We have clarified this point in the revised manuscript (lines 366-373).

Minor issues:

1. Lines 72-74, the sentence "the strategy of depleting intracellular dNTP pools to inhibit viral replication protrudes the convergence of …" is confusing. Did the authors mean " the strategy … illustrates the convergence of …"?

We thank the reviewer for pointing out this ambiguity. We have revised the sentence accordingly. It now reads: "Together, the strategy of depleting intracellular dNTP pools to inhibit viral replication illustrates the convergence of bacterial antiviral defense mechanisms" (lines 72-74).

2. Lines 131-132, "… canonical signaling modules that mediate initiate immune responses …" should be either "… modules that mediate initial … " or "… modules that initiate …".

We thank the reviewer for this helpful suggestion. We have revised the sentence accordingly in the manuscript (lines 130-132: Similarly, we identified fusions of dGTPase domains with the TIR domain, canonical signaling modules that initiate immune responses in both eukaryotes and prokaryotes.)

3. Line 142, "these findings underscore the dGTPase function as versatile immune effector, …". The functions of most dGTPases identified in the bioinformatics screen haven't been experimentally validated. It is unclear whether they act as immune effectors or not. The authors should consider soften the tone, "these findings suggest that …".

We thank the reviewer for this helpful suggestion. We agree that the functions of most dGTPases identified in our bioinformatic screen have not yet been experimentally validated. We have soften the statement accordingly in the revised manuscript (lines 142-144).

4. Line 146-147, "In our analysis of dGTPase-related defense systems, we observed that dGTPase frequently replaces proteins known for their aggregation potential". "replace" is confusing here. Do the dGTPases replace other aggregation-prone proteins at genetic levels? A couple of examples would clarify the issue.

We thank the reviewer for this helpful comment. We agree that the word "replace" was potentially misleading. Our intention was not to imply a demonstrated genetic replacement, but rather that dGTPase can appear in positions analogous to aggregation-prone domains such as SAVED or STING in related domain architectures. To clarify this point, we have revised the text and added examples in the manuscript (lines 146-152).

5. Figure 1c, protein standards with known molecular weight should be marked on the SEC profile for proper evaluation of Vdg size in solution; SEC-MALS would be a more definitive way to determine molecular weight/oligomerization state.

We thank the reviewer for this helpful suggestion. We agree that SEC-MALS provides a more definitive approach to determine the molecular weight and oligomerization state of Vdg. In response, we have performed SEC-MALS experiments and included the results in the revised manuscript (Fig. 2c; lines 189-192), and we have added the corresponding experimental details to the Methods section (lines 494-499).

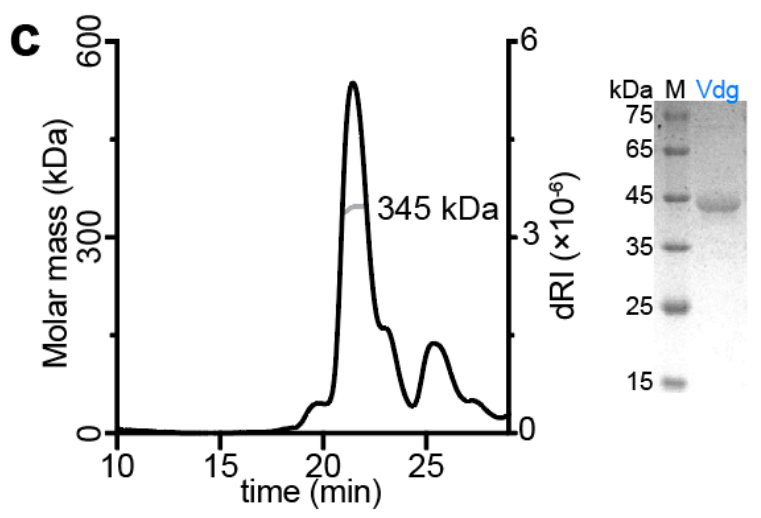

6. Figure 1d, dG and dGTP standards should be included.

We thank the reviewer for this constructive suggestion. In response, we have revised Fig. 2d (corresponding to as Fig. 1d in the reviewer's comment) to include dG and dGTP standards.

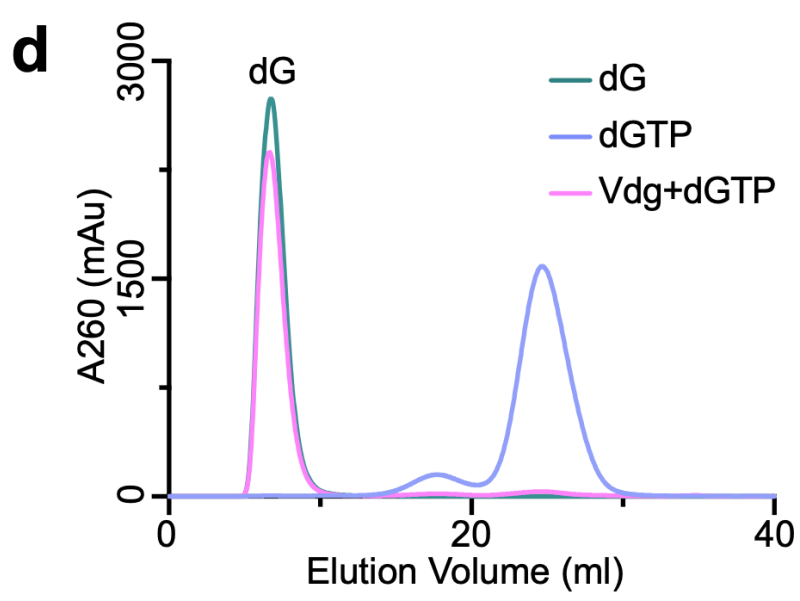

7. Figure 2a, please label molecules A, B, C and D so the readers can place the close-in views in the context.

We thank the reviewer for this insightful suggestion. In response, we have labeled molecules A, B, C, and D in Figure 3a (corresponding to Fig. 2a in the reviewer's comment). We have also added matching annotations in Figure 3b to help readers

place the close-up views in the context of the overall assembly and interaction interfaces.

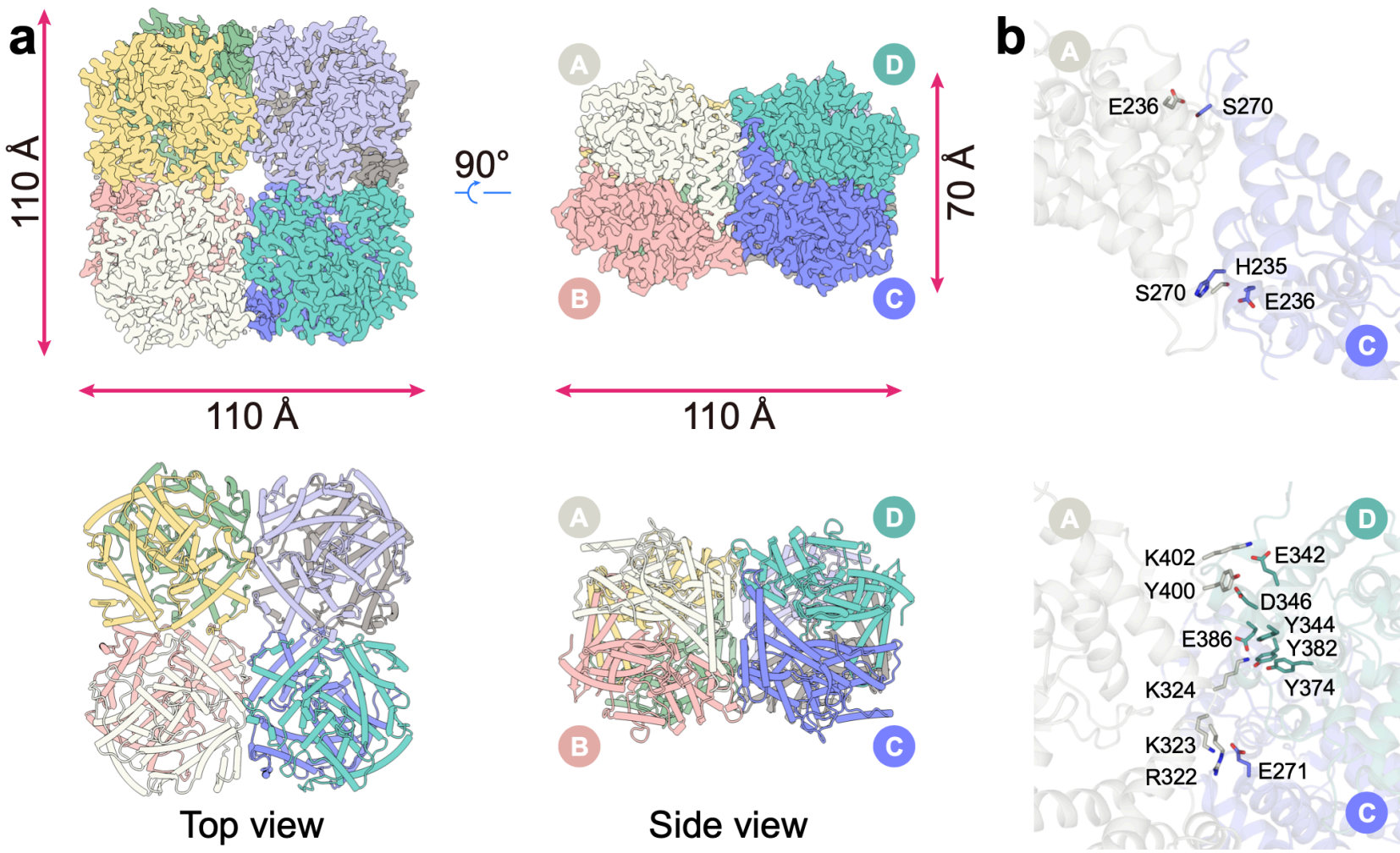

8. In Figure 4b, where are H/A114 and E117? Are they close to the bound dGTP? This information helps understand the active site formation.

We thank the reviewer for raising this important point. In Figure 4b, we have added labels for H/A114 and E117. H/A114 is in close proximity to dGTP, and E117 is positioned nearby as part of the putative catalytic dyad. As noted in the revised text, the catalytic dyad (H125 and E128 in *L. blandensis*) corresponds to H/A114 and E117 in Vdg.

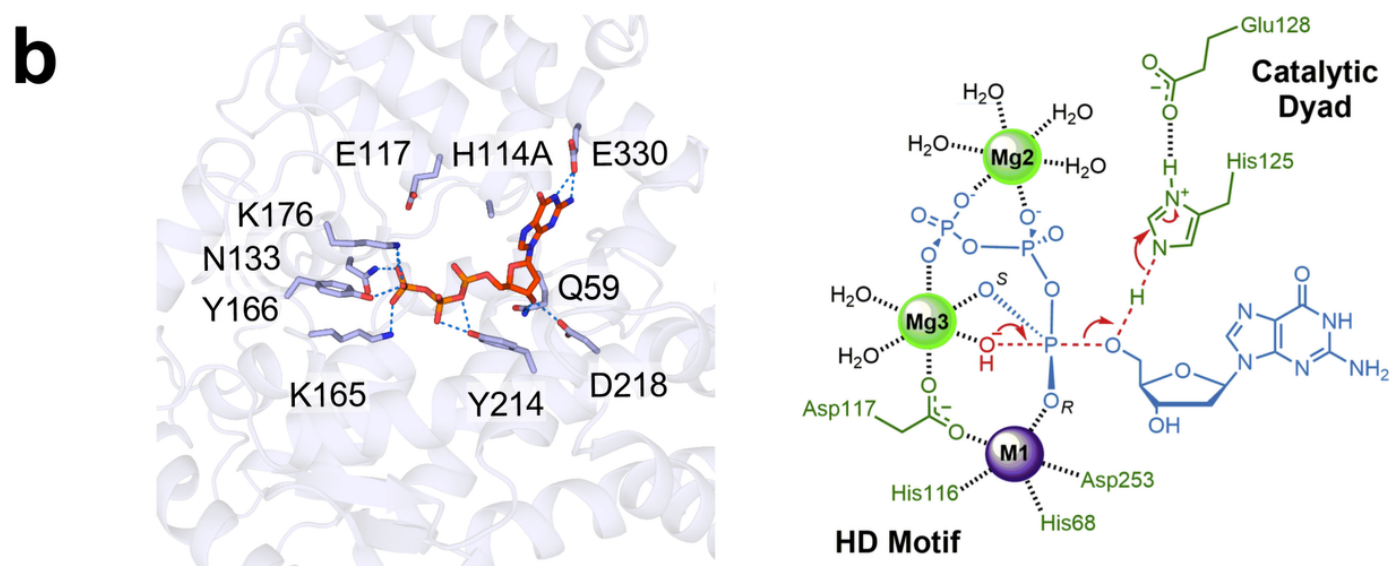

9. Line 292, are the dimer-to-octamer interfacial mutants H235/E236A and R322/K323/K324/Y400/K402 stable in solution? It can be quickly tested with recombinant proteins. It would be interesting to see whether these mutants are stable and still bind dGTP and/or dAMP.

We thank the reviewer for this important concern.

To assess whether the dimer-to-octamer interfacial mutants H235A/E236A and R322A/K323A/K324A/Y400A/K402A are stable in solution, we performed rapid tests using recombinant proteins. Both mutants remained stable under enzymatic reaction conditions (Supplementary Fig. 6g). We also examined whether these mutations affect ligand binding. The mutants retain dGTP hydrolysis activity, consistent with preserved dGTP binding (Supplementary Fig. 2f). In addition, mass spectrometry analysis shows that both mutants retain dAMP binding, similar to wild type Vdg (lines 362-364 and Supplementary Fig. 6g). These results are consistent with the structural observation that the dimer-to-octamer interaction interface is spatially separate from the dGTP and dAMP binding sites (Fig. 4a). We have also incorporated the corresponding experimental details into the Methods section (lines 489-493).

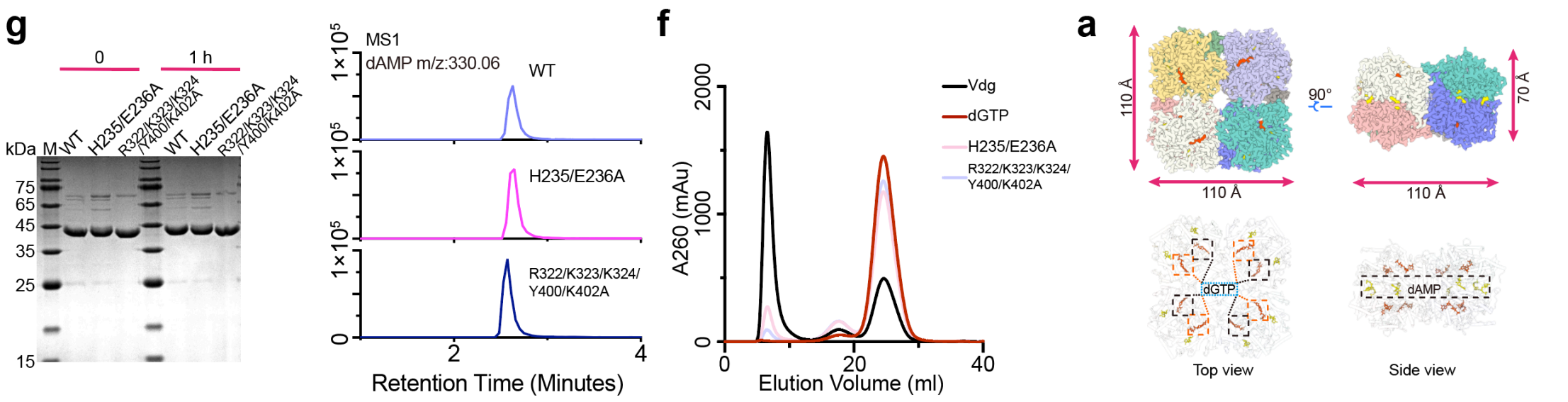

We sincerely thank the reviewers for their time and insightful comments. Below, we provide a detailed, point-by-point response (reviewers comments in red; our responses in black).

****

Reviewer #1 (Remarks to the Author):

The authors have addressed all my concerns. The manuscript is in great shape for publication.

We appreciate the reviewers' positive comments and their recognition that our revisions have further strengthened the manuscript and brought it into excellent shape for publication.

****

Reviewer #2 (Remarks to the Author):

I co-reviewed this manuscript with one of the reviewers who provided the listed reports. This is part of the Nature Communications initiative to facilitate training in peer review and to provide appropriate recognition for Early Career Researchers who co-review manuscripts.

We thank the co-reviewer for the support and insightful feedback throughout the review process.

****

Reviewer #3 (Remarks to the Author):

The authors have successfully addressed all my concerns.

We are grateful for the reviewers' positive feedback and for their acknowledgment that the revisions have strengthened the manuscript.
